# A Machine Reading System for Assembling Synthetic Paleontological Databases

**DOI:** 10.1371/journal.pone.0113523

**Published:** 2014-12-01

**Authors:** Shanan E. Peters, Ce Zhang, Miron Livny, Christopher Ré

**Affiliations:** 1 Department of Geoscience, University of Wisconsin-Madison, Madison, Wisconsin, United States of America; 2 Computer Sciences Department, University of Wisconsin-Madison, Madison, Wisconsin, United States of America; 3 Department of Computer Science, Stanford University, Stanford, California, United States of America; Hellas, Greece

## Abstract

Many aspects of macroevolutionary theory and our understanding of biotic responses to global environmental change derive from literature-based compilations of paleontological data. Existing manually assembled databases are, however, incomplete and difficult to assess and enhance with new data types. Here, we develop and validate the quality of a machine reading system, PaleoDeepDive, that automatically locates and extracts data from heterogeneous text, tables, and figures in publications. PaleoDeepDive performs comparably to humans in several complex data extraction and inference tasks and generates congruent synthetic results that describe the geological history of taxonomic diversity and genus-level rates of origination and extinction. Unlike traditional databases, PaleoDeepDive produces a probabilistic database that systematically improves as information is added. We show that the system can readily accommodate sophisticated data types, such as morphological data in biological illustrations and associated textual descriptions. Our machine reading approach to scientific data integration and synthesis brings within reach many questions that are currently underdetermined and does so in ways that may stimulate entirely new modes of inquiry.

## Introduction

Paleontology is based on the description and classification of fossils, an enterprise that has played out in an untold number of scientific publications. The construction of synthetic databases that aggregate fossil data in a way that enables large-scale questions to be addressed has expanded the intellectual reach of paleontology [1]–[5] and led to fundamental new insights into macroevolutionary processes (e.g., [6]–[9]) and the timing and nature of biotic responses to global environmental change (e.g., [10], [11]). Nevertheless, paleontologists often remain data limited, both in terms of the pace of discovery and description of new fossils and in terms of their ability to synthesize existing published knowledge on the fossil record. Many other sciences, particularly those for which physical samples and specimens are the source of data, face similar challenges.

One of the most successful efforts to compile data on the fossil record to date is the Paleobiology Database (PBDB; http://paleobiodb.org). Founded nearly two decades ago by a small team who generated the first sampling-standardized global Phanerozoic taxonomic diversity curves [12], [13], the PBDB has since grown to include an international group of more than 380 scientists with diverse research agendas. Collectively, this group has spent approximately nine continuous person years entering over 300,000 taxonomic names, 530,000 opinions on the status and classification of those names, and 1.2 million fossil occurrences (i.e., temporally and geographically resolved instances of fossils). Some data derive from the original fieldwork and taxonomic studies of the contributors, but the majority of the data were extracted from approximately 40,000 publications. Nevertheless, the PBDB currently leverages only a small fraction of all published paleontological knowledge, primarily because there is a large and ever-growing body of published work and manually finding and entering data is a labor intensive and often ambiguous task. Moreover, because the end product of manual data entry is a list of facts that are divorced from most, if not all, original contexts, assessing the quality of the database and the reproducibility of results is difficult.

Here we develop and deploy PaleoDeepDive (PDD), a statistical machine reading and learning system, to automatically find and extract fossil occurrence data from the scientific literature. Our motivations for doing so are threefold. First, we aim to test the reproducibility of several key results that are used to frame much of our understanding of the large-scale history of life, including long-term taxonomic diversity and rates of genus-level extinction and origination [1]–[13]. Second, we aim to improve upon the state of the art in machine reading systems, which have not been deployed and validated in a result-focused scientific application. Third, we aim to develop a general machine reading system that has the capacity to change the practice of science by removing substantial time and cost barriers to large-scale data integration and synthesis. In so doing, we hope to shift the balance of effort away from slow and expensive data compilation efforts and towards creative hypothesis testing and the more focused and efficient generation of new primary field- and specimen-based data.

The specific question that motivates this study is: Can the data produced by a machine reading system achieve a quality that is sufficient to enable literature synthesis-based science? We address this question by pitting our system's results against those of human-constructed databases at several levels of granularity, from individual facts that describe opinions on the biological classification of taxa to synthetic results that summarize the history of genus-level biodiversity over millions of years. In all cases, we show that PDD produces data with quality that is comparable to that generated by humans, even when only small amounts of training data are available. We also test the ability of our system to lower the cost of extending a human-constructed database by extracting data from an order of magnitude more references. The results of this experiment show that our system is efficiently scalable and that key macroevolutionary patterns are robust even when derived from different bodies of literature. We further test the ability of our system to incorporate new types of information by extracting morphological data from biological illustrations and their labels, captions, and associated text. Our machine-derived body size estimates are statistically indistinguishable from those produced by humans manually measuring the same illustrations. Because we are focused here on the validation and testing of a new machine reading system, the specific data types and approaches we take are based on many of those taken by humans when building and analyzing the Paleobiology Database. Our system is, nonetheless, designed for broad applicability in the biological and physical sciences, and it can be readily extended for knowledge base creation in many different domains of Earth and life science.

## Materials and Methods: General Description

### Overview

A fundamental challenge faced by machine reading systems is that computers cannot read documents unambiguously. Instead, machines have difficulty with all aspects of document reading, from optical character recognition (OCR) and natural language understanding tasks, to the more complex subtleties involving domain-specific representations of fact. As a result, coping with ambiguity is a key challenge in many areas of computer science [14]–[18].

To accommodate the inherent ambiguity of the scientific literature, PDD is built upon the DeepDive machine reading infrastructure [18], which is designed to extract information in a way that achieves a deep level of contextual understanding. To do this, DeepDive takes a radical approach: it treats all sources of information, including existing data, as evidence that may or may not be correct. Extraction tasks then become probabilistic inference challenges. DeepDive takes a joint or collective probabilistic approach [19], in which all available information is considered simultaneously. This is in contrast to a pipelined approach [17], [20], [21], in which hard decisions are made after each stage of document processing, which can result in compounding errors and suboptimal data quality [22]. DeepDive is also able to accept diverse forms of feedback, including example data sources, formal rules, and training data.

Similar conceptual underpinnings are currently in use by Google's Knowledge Graph, IBM's Watson, and CMU's NELL project. However, none of these have demonstrated an ability to extract information collectively from text, tables, and figures, which is critical to meeting the standards and questions posed by scientific uses. The cost of taking a collective probabilistic approach is that complexity grows exponentially with each new source of ambiguity. Recent work, in part motivated by this study, allows us to perform the requisite statistical inference tasks orders of magnitude more efficiently than was possible just a few years ago [23]–[27].

### PaleoDeepDive Pipeline

**Figure 1:**
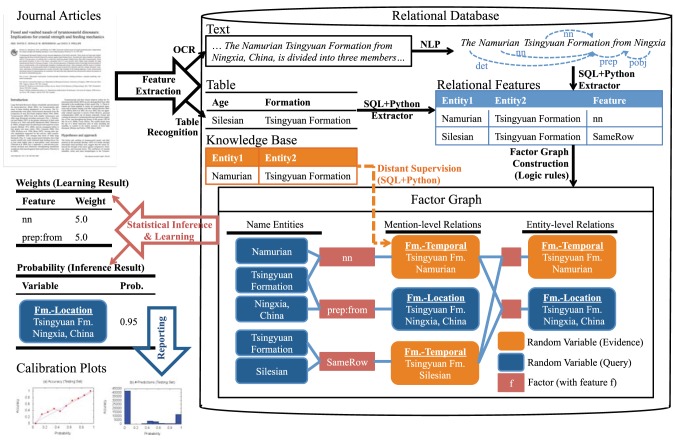
Schematic representation of the PaleoDeepDive workflow. PDF documents (upper left) are subject to Optical Character Recognition (OCR), Natural Language Processing (NLP), table recognition and other third-party software applications that parse and identify document elements. Entities (in this example, geological formations and geographic locations) are identified and related to one another by features (e.g., parts of speech, locations in table), which are recognized and extracted by SQL queries and scripts (e.g., written in Python). Factor graphs are then constructed for entities and possible relationships among them. DeepDive estimates the weight of features based on their expressions in the data. The final step is reporting, which includes explicit probability estimates for each relationship and calibration reports, which can be used to evaluate and improve the system in an iterative fashion.

The input to PaleoDeepDive is a set of documents (e.g., PDFs or HTML), and a database structure that defines entities (i.e., unique instances of specific types of information, such as a specific taxon, time interval, or geological formation) and relationships of interest (i.e., associations between one or more entities). The first step in the DeepDive process is to perform document parsing, including optical character recognition (OCR), document layout recognition, and natural language processing (NLP) of the text (Fig. 1; Figs. S1–S3 in File S1). These steps are required before applying any of the reasoning necessary to recognize entities and the relationships among them. An example of the latter is: “Does this instance of the word ‘Waldron’ refer to the ‘Waldron Shale’, a distinct geological formation, and if so, what is its stated geologic age, where is it located geographically, and which species are reported from it?” Descriptions of how to recognize entities and the relationships among them can be articulated by scientists through rules and examples, which form the basis for specific relational features (e.g., parts of speech, such as “the Waldron Formation is Silurian in age”) that link two or more entities (Fig. 1; Tables S1, S2 in File S1). The weights of these rules are then estimated (i.e., learned) from the data using classical equations based on exponential models [19]. Essentially, the likelihood of the given set of observations (data and rules) is maximized, given the set of features expressed by the rules (Fig. 1).

The end-product of PDD is not a classical database, which consists of isolated facts that are all assumed to be equally correct. Instead, DeepDive produces a probabilistic database in which each fact remains tightly coupled to its original context and is associated with an estimated probability of being correct [28]. A probabilistic approach is not a panacea, but it does allow our system to cope with ambiguity in a principled and consistent way. This is critical for scientists, who can use these probabilities to identify errors and omissions and thereby improve the quality of the system. For further explanation, application code, and example data output see our online documentation (http://deepdive.stanford.edu/doc/paleo.html).

## Materials and Methods: Extended Description

### System

Features that relate facts in PDD are encoded in a relational database. These features derive from two sources: a set of functions written in the DeepDive framework and a set of existing tools developed by other researchers, including Tesseract and Cuneiform for text, Abbyy Fine Reader for tables, and StanfordCoreNLP for linguistic context. The list of features and rules used in this version of PDD are summarized in Tables S1 and S2 in File S1.

After extracting features in documents, the next step is to generate a factor graph (Fig. 1; Fig. S3 in File S1), which is a compact way of specifying exponential family probability models [19], [29]. The factor graph is defined by a hypergraph (V, E) where V is a set of random variables and 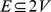 define groups of variables (factors) that are correlated. In addition, each random variable is associated with a domain (for simplicity, consider a Boolean random variable). Each factor (edge) e = (v1,.., vk) is associated with a scalar function called a potential (weight) φe: {0, 1}k→R. For example, the 2-tuple (Tsingyuan Fm, Namurian) represents an ordered pair (i.e., a tuple as defined in set theory) and corresponds to a random variable in DeepDive. This variable assumes the value 1 if true, 0 if false. To specify a correlation, for example, if (Tsingyuan Fm, Carboniferous) is true, then it is likely that (Tsingyuan Fm, Namurian) is also true (because the Carboniferous contains the Namurian), a factor can be encoded to relate the two variables. This factor is only a statistical implication; PDD will estimate the strength of this implication on the data.

The factor graph in PDD can be conceived of as existing in three layers (Fig. 1). The first layer corresponds to the set of entities detected as individual mentions in documents. The second layer corresponds to a set of relation candidates between mentions, and the third layer corresponds to a set of relation candidates between distinct entities. One can think of the second layer as a per document layer and the third layer as the “aggregation” across all documents. Conceptualization of these layers is useful for software engineering reasons, but the statistical apparatus uses information from all layers simultaneously at the inference and learning stages.

Given a factor graph generated by feature extraction, PDD next learns the weight for each factor and then runs inference tasks to estimate the probability of each random variable. One key challenge of machine reading approaches is how to generate training data (i.e., a set of random variables that have been assessed for accuracy and that contain positive and/or negative examples). Traditional approaches include human expert annotation of results and crowd-sourcing [30]. The human-constructed PBDB allows PDD to make extensive use of a generalization of Hearst patterns called distant supervision [31], [32]. This approach to training has considerable potential in the natural sciences because even simple lists of facts, such as the location and general geological age of rock formations, can be used in distant supervision to improve the quality of data extractions and more complex inferences.

Factor graphs are a convenient way to define random variables and their correlations, but they can be large. In PDD, the factor graph contains more than 200 million random variables and 300 million factors with 12 million distinct weights (Table S9 in File S1). PDD uses recent research in both theory [23], [24] and systems [25] to address this computational challenge. Further details are given the Supplementary Information.

### Documents

The serial publications used in the Overlapping Document Set (OverlappingDS) and Whole Document set (WholeDS) are provided in Tables S3 and S8 in File S1. Some of the serials in the top-50 PBDB sources were not accessible to us online. We were also not able to able to recover all references in the listed in the PBDB, regardless of the general online accessibility to the source. This discrepancy is due to incomplete bibliographic information in the human database, which is currently keyed in manually and sometimes in incomplete form (Tables S10, S11 in File S1), and OCR and NLP document processing failures (see Assessment, below). To match retrieved documents to specific PBDB references we first used the TokenSet Cosine similarity approach [33] and then created an Amazon Mechanical Turk job, in which 64 distinct human workers combined for 30,182 evaluations of the matches. To obtain the WholeDS, we extended the OverlappingDS to include all available documents in the top-50 serials in the PBDB; we also included the whole open access Biodiversity Heritage Library.

### Features

All PDD feature extraction tasks that use existing tools were run on Condor and the Open Science Grid (OSG). Ghostscript was run to convert each document into a set of png images. This step is necessary because our approach uses both the content of text and the information conveyed by the detailed formatting and layout of elements within and adjacent to that text (e.g., font, justification, figure elements etc.). Next, OCR tools were executed. Each tool was permitted to run for 24 hours on a document before timeout occurred; a failed document was re-deployed on the OSG up to 10 times before being removed from the set. Document failures were caused by kernels older than 2006 and incompatible software on individual OSG machines, as well as document-specific software bugs, such as segmentation faults in Cuneiform caused by unusual document formatting. All tools had a failure rate of less than 8%, but these errors are orthogonal to our work; future improvements to OCR and NLP tools will improve the quality of PDD.

The WholeDS contains 23 times more documents than the OverlappingDS, and the number of variables extracted from them scales approximately linearly. The number of distinct features is, however, only 13 times greater because features can be shared across documents (Table S12 in File S1). Distinct taxa are only 10 times more numerous in the WholeDS because many taxa are referred to in more than one document. The number of occurrences is only six times greater in the WholeDS, reflecting the fact that most of the additional documents we were able to access are taxonomically-focused and do not contain fossil occurrence data; some documents also derive from serials, such as USGS Open-File Reports, that are interdisciplinary, with only a minority of documents that are relevant to paleontology.

### Extensions

We extended PDD to include data extraction from German and Chinese language documents. The named entity recognition component of PDD has dictionary-based features and NLP-based features. Relevant language-specific dictionaries were built manually and from external sources such as geonames.org. For NLP-based features, the Stanford CoreNLP provides models for Chinese and German. For document layout-based features, there is no change in function with language.

We also extended PDD to extract body size from biological illustrations, which requires processing images, linking image part labels to captions, and mapping captions to text in order to extract all of the necessary information (Fig. S9, S10 in File S1). Further explanation of tools and methods used for joint image-text analysis is presented in the Supplementary Information.

## Results

### Overlapping Document Set (OverlappingDS)

To quantitatively assess PDD's ability to read the literature and extract structured fossil occurrence data, we used the human-constructed PBDB as a baseline for comparison. Specifically, 11,782 documents from the top-50 serial publications in the PBDB were also processed by PDD (the OverlappingDS; Table S3 in File S1; File S2). This experiment allows comparisons to be made between human readers and our system at multiple levels of granularity. Because PDD depends on linguistic understanding, at this time our system is able to process only English, German, and Chinese language documents, which constitute 76%, 6%, and 2%, respectively, of PBDB's total reference inventory. Additional languages will be added to the system as new NLP and OCR software for processing these languages become available.

On average, PDD extracts more taxonomic data from a document than humans. For example, humans extracted 79,913 opinions on the status and biological classification of taxonomic names from the OverlappingDS, whereas PDD extracts 192,365 opinions. Although many of these opinions are relatively simple cases that are often not entered by humans (e.g., a species belongs to a genus), they nonetheless constitute taxonomic information which is sometimes not entered by humans at all. For example, PDD extracted 59,996 taxonomic names from the OverlappingDS that were never formally entered by human readers from any of the over 40,000 references they have entered thus far. A random sample of these names indicates that most are valid species-level taxa and that ≥90% were correctly extracted (Table S4 in File S1). The cases where PDD fails to recognize and extract data from a document are due primarily to OCR-related errors (Tables S5, S6 in File S1), which are orthogonal to this work.

The quality of PDD's database was assessed in three ways. The first uses DeepDive's pipeline, which produces internal measures of precision for every entity and relationship. All of the extractions used here have a precision of ≥95% according to this criterion (like all p-value thresholds, the decision to use 95% is arbitrary). We also conducted blind assessment experiments of two types. In the first double blind experiment, we randomly sampled 100 taxonomic opinions from the PBDB and PDD and then randomized the combined 200 opinions into a single list. This list was then manually assessed for accuracy relative to the source document. In this assessment, PDD achieved ≥92% accuracy in all cases, which is greater than or equal to the accuracy estimated for the human data (Table S7 in File S1). In the second blind experiment, eight scientists with different levels of investment in the PBDB were presented with the same five documents and the same 481 randomly selected taxonomic opinions, which were extracted by both humans and PDD (Fig. S4 in File S1). No indication was given regarding which system generated the facts. Humans measured a mean error frequency in the machine-constructed database of 10%, with a standard deviation of ±6%. This is comparable to the error rate of 14±5% they estimated for those same documents in the human-constructed database (Fig. S5 in File S1). Variability in estimates between annotators reflects a combination of assessment error and divergent interpretations of what constitutes a taxonomic opinion in the literature. Although the blind experiments suggest that the error rate is comparable between the databases, the comparisons are not strictly equivalent. For example, PDD currently understands only nested hierarchical relationships (i.e., genus a belongs to family b) and synonymy (genus a is a junior synonym of genus b), which comprise a large fraction (90% and 5%, respectively) but not all of the taxonomic opinions in the PBDB (other opinions include *nomen dubia*, *nomen nuda*, and other formal opinions on nomenclatural status defined by the International Codes of nomenclature, namely the ICZN and IAPT). Another reason that our results are not strictly comparable is that human data enterers often selectively enter only the data that they deem to be important or non-redundant with data in other documents. This selectivity occurs primarily because the manual data entry process is time consuming, which causes decisions to be made on the basis of necessity, not exhaustiveness.

**Figure 2:**
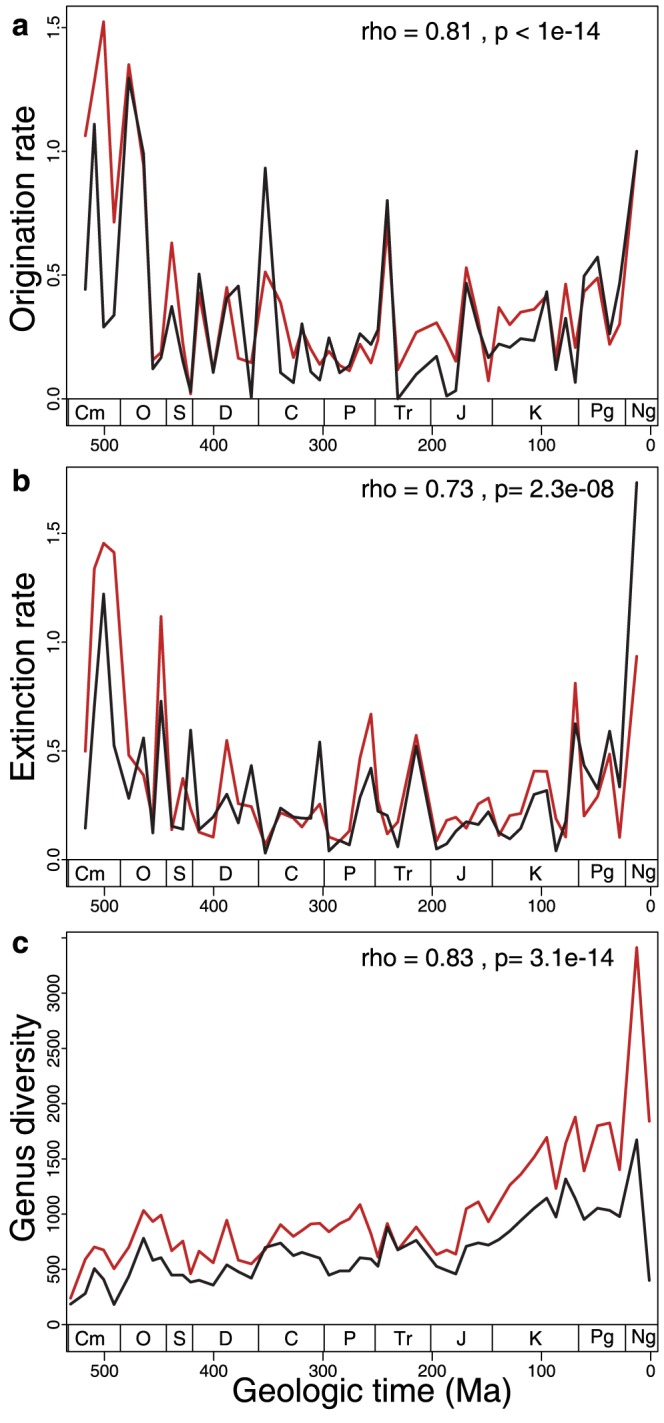
Machine- and human-generated histories of taxonomic diversity and rates of genus-level turnover. Data derive from reading of the overlapping document set. Human-generated in red, machine-generated in black. Spearman rank order correlations for time series that have been detrended by taking first differences shown. (**a**) Per capita, per interval origination rates [34]. (**b**) Per capita, per interval extinction rates. (**c**) Total range-through diversity. Data for analyses accessible in File S3.

**Figure 3:**
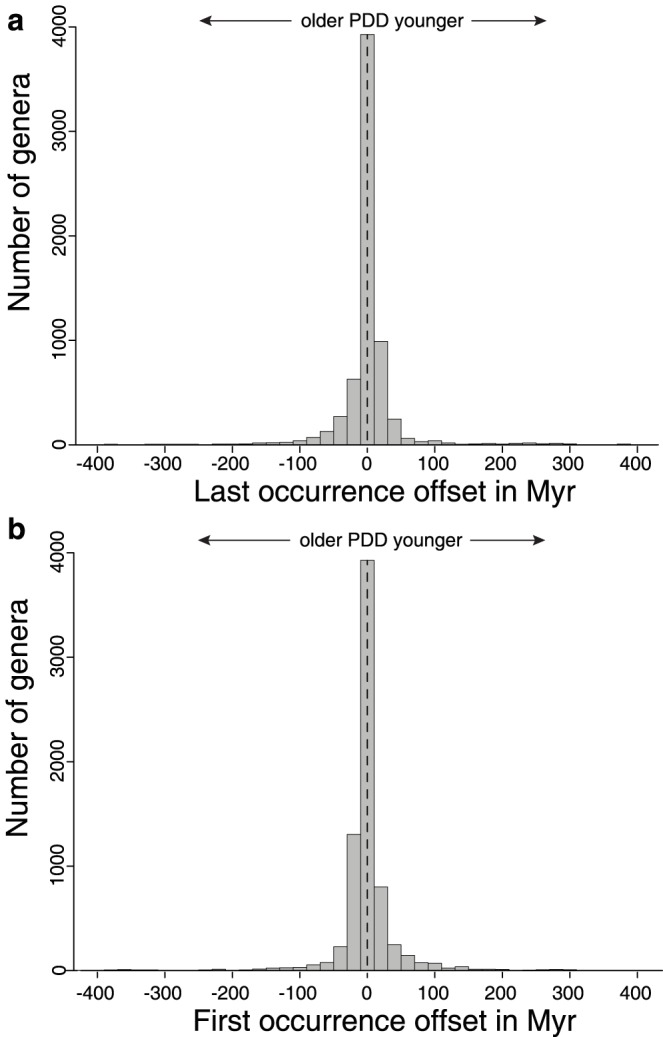
Genus range offsets in human and machine-generated data. Results for 6,708 genera common to the PBDB and PDD in the OverlappingDS are shown. (**a**) Last occurrence differences. Median is 0 Myr, mean is +1.7 Myr. (**b**) First occurrence offset. Median is 0 Myr, mean is −0.3 Myr. Data for analyses accessible in File S4.

The third approach we took to assessing PDD quality was conducted at the aggregate level of Phanerozoic patterns of taxonomic diversity and rates of genus-level taxonomic turnover [34]. After processing both databases with the same algorithms to generate a working taxonomy and a list of occurrences meeting the same threshold of temporal resolution (i.e., epoch or finer), we find good overall agreement in results (Fig. 2; data are binned into the same 52 time intervals, mean duration 10.4 Myr). Both long-term trends and interval-to-interval changes in genus-level diversity and turnover rates are strongly positively correlated, indicating that both databases capture the same underlying signal. The number of genus-level occurrences in each time interval, which is important to sampling standardization approaches [35], [36], are also positively correlated (for time series that have been detrended by taking first differences, Spearman rho = 0.65; p = 5.7×10^−7^). The times of first and last occurrence of 6,708 taxonomically and temporally resolved genera common to both database are also consistent statistically, although there are large range offsets owing to errors in both the human- and machine-generated databases (Fig. 3).

Differences between results (Fig. 2) can be attributed to a combination of errors and inconsistencies in the human-constructed database, as well as to data recovery and inference errors committed by PDD. For example, the PBDB contains typographical errors introduced during data entry. But, most of the differences observed in Fig. 2 are attributable to more insidious inconsistencies. For example, there are groups of occurrences in the PBDB that derive from multiple documents, even though only one document is cited as their source. Thus, the actual number of references used to generate the PDD results is larger than that used by PDD. Occurrences in the PBDB are also sometimes attributed to a reference that actually contains no data but that instead cites the PBDB or some other archive that we did not access as its data source. A much more common source of discrepancy involves the injection of facts and interpretations by humans during the data entry process. Notably, approximately 50% of the ages assigned to fossil occurrences in the human database are not actually mentioned in the cited reference (Fig. S6 in File S1). Although problematic in some senses, this is well justified scientifically. The stated age for an occurrence in a document is often not the best available age, and the PBDB has no capacity to dynamically assign ages based on all evidence. Humans attempt to account for these structural limitations in the database by entering what they determine, on the basis of other evidence, to be the best age for a fossil occurrence in a document. PDD replicated aspects of this behavior by inferring across all documents the most precise and recently published age for a given geological unit and location, but this is not sufficient to cover the full range of sources consulted by humans. Thus, a disproportionate number of the occurrences extracted by PDD have a temporal resolution that results in their exclusion from the quantities shown in Fig. 2 (e.g., geological period-level). Including occurrences with low temporal resolution causes the absolute values of the human- and machine-generated diversity curves to converge (Fig. S7 in File S1).

Errors and limitations in the current PDD system also account for divergence in results (Fig. 2). For example, OCR-related document processing failures, often involving tables, are among the leading causes of errors of omission by PDD (Table S6 in File S1). The current version of PDD also has design elements that cause some facts to be omitted. For example, PDD currently places great importance on formal geologic units, which means that no fossil occurrences are recognized in references that do not have well defined geologic units. Because this situation is more prevalent in recent time intervals, the lower total diversity recovered by PDD towards the recent (Fig. 2) is attributable to this design decision. Omissions also occur when a fact is correctly extracted by PDD, but with a probability <0.95, the arbitrarily chosen probability threshold used here. This type of confidence-related error can typically be overcome by defining new features or rules, such as natural language expressions, that can be used to remove sources of ambiguity and improve statistical confidence in correct extractions.

The results from the OverlappingDS experiment demonstrate that our system performs comparably to humans in many complex data extraction and inference tasks and that patterns of taxonomic diversity and genus-level taxonomic turnover are similarly expressed in both databases. This is an important result that demonstrates the robustness of widely-used macroevolutionary results and that addresses several long-standing challenges in computer science. However, it is also the case that these specific quantities, which are based on large numbers of taxa, are often robust to random errors introduced at the level of individual facts [37]–[39]. Thus, our results (Fig. 2) could be interpreted as evidence for the presence of a strong signal in the paleontological literature that is readily recovered, regardless of approach. The rather narrow distribution of range offsets on a per-genus basis (Fig. 3), however, suggests that PDD's precision is high even at the scale of individual facts. Note, however, that for many questions requiring that lineage first and last occurrences are known with the highest possible precision, there is still discrepancy between PDD and the human database (Fig. 3).

### Training Data Requirements

We used the human-constructed PBDB as both a source of training data and as a benchmark for evaluation. Therefore, an obvious question is, how big would the human database have to be in order for there to be sufficient training data to obtain a high quality result?

**Figure 4:**
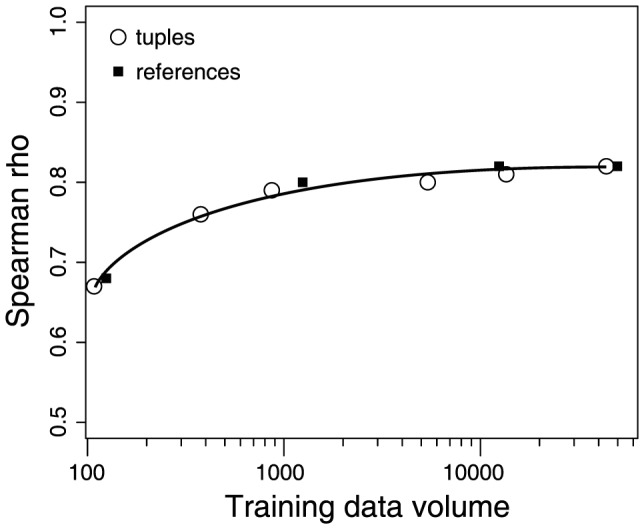
Effect of changing PBDB training database size on PDD quality. Spearman rho is correlation between human- and machine- generated time series of diversity, as in Fig. 2c. Tuples refers to the number of human-constructed relationships between entities (i.e., the Waldron Formation is Silurian in age) that were used for distant supervision. References refers to the number of individual published papers that were read and processed by human and then used for distant supervision. Each curve was generated by subsampling to the specified number of human-processed references and tuples used for distant supervision.

To assess the effect of training data volume on the quality of PDD, we randomly sampled the human database to produce a series of smaller databases. We then re-ran the entire PDD system in exactly the same way, but using only the subsampled data for training purposes. As expected, both the amount of data extracted by PDD (with a probability ≥0.95) and the accuracy of those data, summarized as the Spearman rank-order correlation between first differences in genus-level diversity (Fig. 2c), increases with the amount of training data. However, rather little training data is required in order to achieve a similarly high-quality result (Fig. 4). If the PBDB were populated with just 2% of the total number of references entered by humans over nearly two decades, there would be sufficient training data to obtain a comparable result.

### Additional Assessment

The OverlappingDS was randomly and evenly split into a training set and a testing set. Fifty documents in the testing set were then randomly sampled for assessment by human annotators, primarily graduate students in the Dept. of Geoscience at UW-Madison. Assessments included taxonomic, stratigraphic, chronologic, and geographic tuples. PDD achieves ≥92% human-estimated accuracy in all relations (Table S13 in File S1), which is close to the 95% confidence threshold specified for data output.

The number of facts recovered vs. the number of facts contained in a document (i.e., recall) is more difficult to assess than the precision of the data that are extracted. Because each extracted relationship consists of a paired object and subject (e.g., the object “formation” contains a subject “taxon”), one basic measure of recall is the fraction of all subjects in the PBDB that PDD also recovered. This estimate of recall ranges from 21% to 69%, depending on relation (Table S13 in File S1). For the lowest recall relations, we randomly sampled 10 documents in order to compare the PBDB and PDD. We did so for a combination of three binary relations (taxon,formation)(formation,temporal)(formation,location). When summarizing this 4-part tuple by projecting these relationships to taxon, approximately 18% of PDDs extractions also appear in PBDB and 11% of PBDB extractions also appear in PDD. This implies that both PDD and PBDB make recall errors, but that both systems also have high precision.

Further examination of PDD recall errors (Table S6 in File S1) shows that they can be attributed to OCR-related failures (56%), table recognition problems (29%), and lack of context features that are required to address the full range of often complicated expressions in the literature (15%). All of these errors correspond to interesting and open-problems for computer science. The first two are related to data acquisition (i.e., how to correctly recognize the structure and content of a given document), and the latter is an important natural language inference problem (i.e., how to extract relations by taking advantage of information in the whole document). Continued work in these areas will further improve the PDD system, which we have shown is now capable of meeting, and in some cases exceeding, human standards in its ability to produce a synthetic database resource with scientific value. For additional technical validation of the system, including an explanation of the calibration of probabilities in the database (Figs. S11, S12 in File S1) and the impact of including rich features on overall system quality (Figs. S13, S14 in File S1), see the Supplementary Information.

### Whole Document Set (WholeDS)

**Figure 5:**
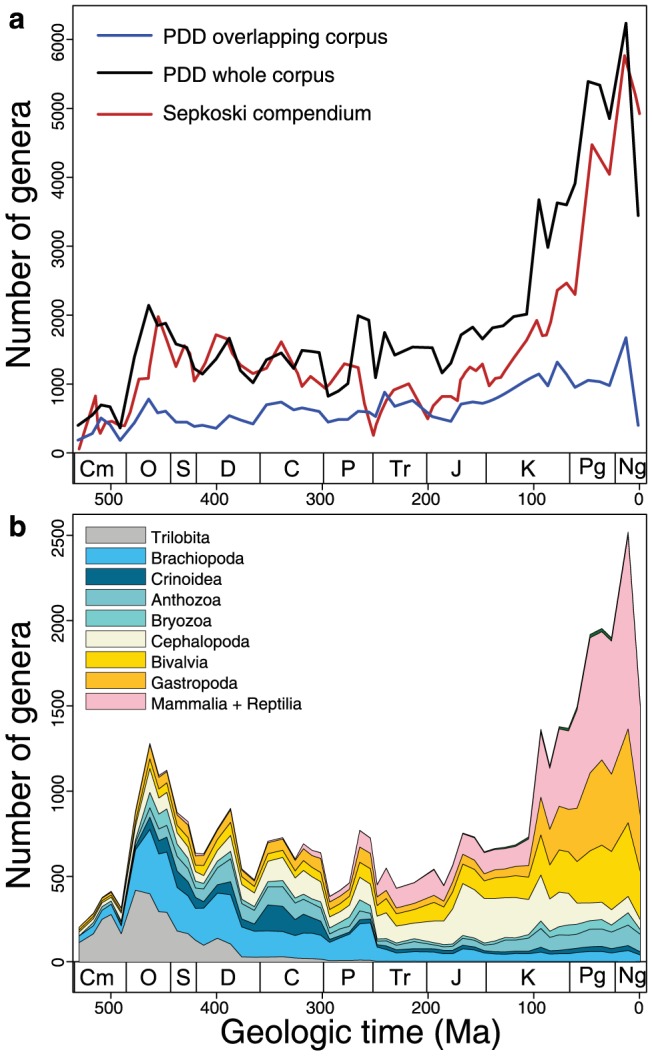
Machine-generated Phanerozoic diversity curves. Genus-level diversity generated by PDD for the whole document set. (**a**) Total genus diversity calculated as in Fig. 2. For comparison, Sepkoski's genus-level diversity curve [3], [4] is plotted using his stage-level timescale. (**b**), Diversity partitioned by genera resolved to select classes by PDD. Data for analyses accessible in File S5.

Scaling PDD up to extract data from every relevant published document poses little technical challenge [40] and would offer a statistical advantage that could improve the overall quality of our system. However, access to the scientific literature for the purpose of automated text and data mining is currently limited [41]. Thus, PDD's entire document set now consists of only 294,463 documents (Table S8 in File S1). Notably for this study, many of these documents were obtained from the open-access Biodiversity Heritage Library, which contains a large number of valuable but also older and taxonomically-focused publications that lack fossil occurrence data used to generate Figs. 2 and 5.

Despite limitations on our ability to access much of the relevant paleontological literature, the PDD-generated Phanerozoic diversity curve for the WholeDS (Fig. 5) yields a face-value empirical genus diversity history that is consistent with classical estimates [3], [4]. First differences in Phanerozoic diversity extracted from the WholeDS are also positively correlated with first differences in diversity for the whole PBDB (Table 1). Genus-level rates of extinction and origination are also similar in both compilations (for first differences, p<0.0004). The diversity histories of major groups of organisms comprising this total diversity are also positively correlated (Table 1), even though fewer than 25% of the references in the PBDB were read and processed by PDD (a total of 22,250 valid genera with resolved stratigraphic ranges are common to both compilations).

**Table 1:** Correlations between human- and machine-generated genus diversity.

Taxonomic group	Spearman rho	P-value
All genera	0.72	3.6×10^−9^
Bivalvia	0.67	6.2×10^−8^
Bryozoa	0.64	3.6×10^−7^
Gastropoda	0.59	5.3×10^−6^
Anthozoa	0.53	6.6×10^−5^
Brachiopoda	0.52	0.0001
Reptilia	0.50	**0.0002**
Trilobita	0.49	0.0003
Cephalopoda	0.41	0.003
Mammalia	0.40	0.004
Crinoidea	0.39	0.004

Data derive from the whole document set and the entire PBDB. Spearman rank-order correlation coefficients and p-values for detrended diversity time series (from Fig. 5b) shown.

## Discussion

The results of our validation study have three important implications. First, we have demonstrated that our machine reading system is capable of building a structured database from the heterogeneous scientific literature with quality that is comparable to a database produced by humans manually reading and extracting data (at least in the dimensions addressed here). This is notable because current benchmarks in machine reading and knowledge base construction, such as the Text Analysis Conference Knowledge Base Population competition, achieve less than 50% accuracy (albeit in the broader domain of general web text). Second, we have tested at a large scale the reproducibility of the PBDB, and in so doing we have identified sources of error and inconsistency that have a bearing on the use of the database. However, we have also shown that key macroevolutionary results are robust to these types of errors. Third, we have shown more broadly that literature-based macroevolutionary patterns are similarly expressed even when they derive from different bodies of literature. This indicates that the paleontological literature, and presumably the underlying fossil record that it has sampled, contains a strong macroevolutionary signal that is readily recovered. This does not mean that our understanding of the global fossil record is uniformly complete taxonomically, temporally, or spatially (Fig. S8 in File S1), that our understanding of the true history of global biodiversity is accurate [12], [13], [42], [43], or even that the literature contains accurate data for every clade (e.g., [39]). It is also the case that the PBDB, our analysis, and many paleobiological analyses to date, have focused on the operational units of Linnean taxonomy, which in some cases yield results that are inconsistent with those that also incorporate phylogenetic approaches and hypotheses (e.g., [44]–[46]).

The ability to expand existing databases and to more rapidly create new high quality synthetic data resources is a notable advance in the methodological toolkit of scientists. However, a much greater advantage of our approach is that the type of database that it produces is fundamentally different from manually populated databases. In the probabilistic database [28] produced by PDD, every fact is associated with an estimated probability of being correct and each fact remains tightly coupled to its original context. Thus, the quality of the entire database can be improved systematically whenever feedback is given on any one component or when additional rules or data is added to the system. More importantly, PDD's data acquisition process is based on the visual and textual analysis of entire documents. Our system is, therefore, able to recognize and extract data that are not currently part of a database but that are contextually related.

For example, the illustration of specimens is central to biological systematics and there are millions of biological illustrations in the WholeDS. Body size, a fundamental property of organisms that determines many aspects of their ecology (e.g., [47]), is one of the morphological attributes readily conveyed by illustrations and their associated text. Several studies have examined the evolution of body size in individual lineages (e.g., [6], [48]–[50]), but, similar to the PBDB, all efforts to manually compile body size data cover only a small portion of the literature and yield monolithic databases that are difficult to assess and extend with new data.

**Figure 6:**
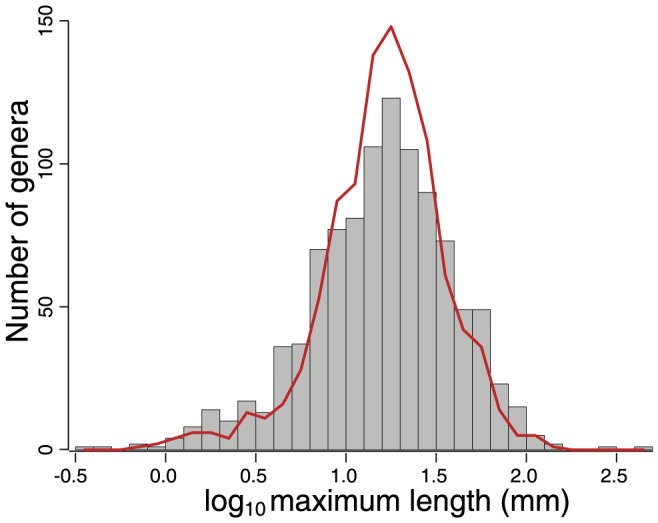
Machine- and human-generated body size estimates. A total of 1,014 brachiopod genera are shown. PDD, gray bars; human estimate, red line. Distributions not significantly different according to paired Mann-Whitney U-test (p = 0.18) and Kruskal-Wallis test (p = 0.64).

To test the ability of our machine reading and learning system to incorporate data in illustrations, we extended PDD to identify images of biological specimens, locate and measure their major and minor axes, and read associated figure labels, captions, and text in order to determine magnification, the portion of the organism being imaged, and taxonomy (see Supplementary Information). The PDD-estimated body sizes for classified brachiopod genera are congruent with body sizes estimated for those same genera by the manual measurement of images (Fig. 6). Leveraging PDD's capacity to quantitatively analyze the entire body of published biological illustrations, in the context of their full textual descriptions, will enable new approaches to biological systematics and morphometrics and brings within reach questions that require a combination of morphological, geologic, and taxonomic data. Before PDD can be deployed to leverage this new capability, the current barriers to automated access and processing of published scientific documents must be overcome. These barriers include the outright prohibition of automated accessing and processing of documents for any purpose, even in cases where the originating institution has paid for online subscriptions, and publisher-imposed limitations on what can and cannot be done with the data that are extracted by machine reading approaches [41]. In those cases where permission is not required because documents are open access, programmatically accessing large numbers of journals typically requires writing customized scripts for each source, which may decide to change accessibility protocols; there are also few standards adhered to between publishers, which adds complexity to the process of accessing digital publications en masse.

Although we have focused here on validating PDD and on testing the robustness of literature-derived taxonomic diversity and turnover patterns in a widely used human-constructed database, our approach is built upon on a general machine reading and learning system [18] that can be readily adapted for many different domain-specific data extraction and inference tasks. For example, many paleobiologists and biologists expect phylogenetic approaches to replace many of the analyses that have traditionally been based on Linnean taxonomic units [44]–[46]. Nevertheless, our machine reading system could be modified to accommodate different data types, including character data and phylogenetic hypotheses (i.e., the results in cladograms and other unranked cladistic summaries of inferred evolutionary relationships). Thus, the specific choice of data focused on here is less important than the fact that we have demonstrated the ability to extract those data with high quality. We have also shown that voluminous training data are not necessarily required to achieve high quality results, though it is always the case that more data will improve statistical inference and overall recall and precision. Thus, many questions that have been posed before, but that have been deemed too difficult to address without prohibitively time consuming data compilation efforts, are now coming within reach. Perhaps more importantly, our approach to data synthesis yields a probabilistic database that remains tightly coupled to primary sources, that continually improves with the addition of new information, and that is capable of integrating complex data in ways that are may stimulate entirely new modes of inquiry.

## Supporting Information

File S1 **Figure S1 in File S1 Schematic representation of the PDD workflow.** **Figure S2 in File S1 Overview of PDD feature extraction.** Text, tables, and images in an original document are parsed (e.g., by table position extraction or natural language). Two or more entities and the specific properties in the document (i.e., features) that relate them are expressed as a row in a database. **Figure S3 in File S1 Overview of factor graph component of PDD.** Existing knowledge bases, such as data in the PBDB, are used to assess mention-level relations during distant supervision. Variables assessed for accuracy become evidence variables for statistical inference and learning steps. **Figure S4 in File S1 Screen shot of web user interface used in blind experiment conducted by 7 human annotators.** A unique link and instructions to complete the form were emailed to each participant, along with written instructions describing the nature of the task. **Figure S5 in File S1 Summary of results of annotation experiment of PDD and PBDB taxonomic extractions.** Human volunteers with varying levels of professional investment in the PDD were asked to assess a mixture of human- and machine-extracted data. **Figure S6 in File S1 Summary of results of annotation experiment of occurrence data.** Human volunteers were asked to assess human-generated occurrence data in the PDD. Although no indication was given as to the source of the data, many of the annotators assumed that they were evaluating machine-generated data. **Figure S7 in File S1 PDD genus-level diversity (black curve) calculated using occurrences with period level or finer temporal resolution.** The results in this figure can be compared to the epoch or finer temporal resolution used to generate the results in Fig. 2c. **Figure S8 in File S1 Geographic distribution of occurrences in PDD-generated database.** Geographic coordinates were extracted for each fossil occurrence extracted by PDD, primarily by identifying location entities linked to geological formations that are also in open georeferenced libraries. **Figure S9 in File S1 Image processing component for body size extraction.** **Figure S10 in File S1 Relation extraction component for body size extraction.** **Figure S11 in File S1 Calibration plots for all relations in OverlappingDS.** **Figure S12 in File S1 Calibration plots for all relations in WholeDS.** **Figure S13 in File S1 Lesion study of deep NLP features and table recognition.** Here we show the results of removing specific components used in recognizing features. **Figure S14 in File S1 Lesion study of joint inference.** Here we explore the quantitative effects of disabling specific joint inference capabilities. **Table S1 in File S1 List of features and rules used in the current version of PDD.** Finding the right simple features and rules can be difficult. The PDD system is designed to operate in an iterative fashion, with error analysis occurring after each round of feature and rule definition. **Table S2 in File S1 List of distant supervision rules used in PDD.** **Table S3 in File S1 Distribution of documents in the OverlappingDS.** **Table S4 in File S1 Error Analysis of taxon entity extractions in PDD.** **Table S5 in File S1 Error analysis of all PDD extractions.** **Table S6 in File S1 Error analysis of all PBDB extractions.** **Table S7 in File S1 Comparison of Accuracies of PDD and PBDB.** **Table S8 in File S1 Statistics for WholeDS.** **Table S9 in File S1 Factor graph statistics in the overlapping and whole document sets.** **Table S10 in File S1 Random sample and assessment of PBDB journal articles from the** ***Journal of Vertebrate Paleontology*** **not appearing in the OverlappingDS.** **Table S11 in File S1 Random sample and assessment of PBDB journal articles from the** ***Science*** **not appearing in the OverlappingDS.** **Table S12 in File S1 Extraction statistics for the OverlappingDS and WholeDS.** **Table S13 in File S1 Statistics of PDD annotations and quality score for each relation.**
(PDF)

File S2 **Comma separated value file of fossil occurrences extracted by PDD for OverlappingDS.**
(CSV)

File S3 **Comma separated value file of raw data used in PDD-PBDB taxonomic diversity analyses in OverlappingDS.**
(CSV)

File S4 **Comma separated value file of raw data used in PDD-PBDB genus range offset analyses.**
(CSV)

File S5 **Comma separated value file of raw data used in PDD-PBDB taxonomic diversity analyses in WholeDS.**
(CSV)

## References

[1] Raup DM (1976) Species diversity in the Phanerozoic: a tabulation. Paleobiology 2:279–288.

[2] Bambach RK (1977) Species richness in marine habitats through the Phanerozoic. Paleobiology 3:152–167.

[3] Sepkoski JJ Jr (1981) A factor analytic description of the Phanerozoic marine fossil record. Paleobiology 7:36–53.

[4] Sepkoski JJ Jr (1998) Rates of speciation in the fossil record. P Trans R Soc B 353:315–326. 10.1098/rstb.1998.0212 PMC1692211 11541734

[5] Benton MJ (1995) Diversification and extinction in the history of life. Science 268:52–58. 7701342 10.1126/science.7701342

[6] Alroy J (1998) Cope's rule and the dynamics of body mass evolution in North American fossil mammals. Science 280:731–734. 9563948 10.1126/science.280.5364.731

[7] Jablonski D, Roy K, Valentine JW (2006) Out of the tropics: evolutionary dynamics of the latitudinal diversity gradient. Science 314:102–106. 17023653 10.1126/science.1130880

[8] Kiessling W (2005) Long-term relationships between ecological stability and biodiversity in Phanerozoic reefs. Nature 433:410–413. 15674290 10.1038/nature03152

[9] Alroy J (2010) The shifting balance of diversity among major marine animal groups. Science 329:1191–1194. 20813951 10.1126/science.1189910

[10] Finnegan S, Heim NA, Peters SE, Fischer WW (2012) Climate change and the selective signature of the Late Ordovician mass extinction. P Natl Acad Sci USA 109:6829–6834. 10.1073/pnas.1117039109 PMC3345012 22511717

[11] Blois JL, Zarnetske PL, Fitzpatrick MC, Finnegan S (2013) Climate Change and the Past, Present, and Future of biotic interactions. Science 341:499–504. 23908227 10.1126/science.1237184

[12] Alroy J, Aberhan M, Bottjer DJ, Foote M, Fürsich FT, et al (2008) Phanerozoic trends in the global diversity of marine invertebrates. Science 321:97–100. 18599780 10.1126/science.1156963

[13] Alroy J, Marshall CR, Bambach RK, Bezusko K, Foote M, et al (2001) Effects of sampling standardization on estimates of Phanerozoic marine diversification. P Natl Acad Sci USA 98:6261–6266. 10.1073/pnas.111144698 PMC33456 11353852

[14] Ferrucci DA, Brown E, Chu-Carroll J, Fan J, Gondek D, et al (2010) Building Watson: an overview of the deepqa project. AI Magazine 31:59–79.

[15] Murphy K (2013) From big data to big knowledge. In: Proceedings of the 22nd ACM international conference on Conference on information & knowledge management, CIKM'13. New York, ACM. pp. 1917–1918.

[16] Suchanek FM, Sozio M, Weikum G (2009) Sofie: A self-organizing framework for information extraction. In Proceedings of the 18th International Conference on World Wide Web, WWW'09. New York: ACM. pp. 631–640.

[17] Carlson A, Betteridge J, Kisiel B, Settles B, Hruschka ER Jr, et al**.** (2010) Toward an architecture for never-ending language learning. In AAAI.

[18] Kumar A, Niu F, Ré C (2013) Hazy: making it easier to build and maintain big-data analytics. Commun. ACM 56:40–49.

[19] Getoor L, Taskar B (2007) Introduction to Statistical Relational Learning. Cambridge: The MIT Press. 608 p.

[20] Krishnamurthy R, Li Y, Raghavan S, Reiss F, Vaithyanathan S, et al (2009) Systemt: a system for declarative information extraction. SIGMOD Rec. 37:7–13.

[21] Li Y, Reiss F, Chiticariu L (2011) Systemt: A declarative information extraction system. In ACL (System Demonstrations), pp. 109–114.

[22] Govindaraju V, Zhang C, Ré C (2013) Understanding tables in context using Standard NLP toolkits. ACL 2:658–664.

[23] Niu F, Recht B, Ré C, Wright SJ (2011) Hogwild: a lock-free approach to parallelizing stochastic gradient descent Advances in Neural Information Processing Systems. 24:693–701.

[24] Liu J, Wright SJ, Ré C, Bittorf V, Sridhar S (2014) An asynchronous parallel stochastic coordinate descent algorithm. Proceedings of the 31st International Conference on Machine Learning JML 32.

[25] Zhang C, Ré C (2013) Towards high-throughput Gibbs Sampling at scale: A study across storage managers. SIGMOD ' 13:397–408.

[26] Recht B, Ré C (2012) Toward a noncommutative arithmetic-geometric mean inequality: conjectures, case-studies, and consequences. JMLR: Workshop and Conference Proceedings 23:11.1–11.24.

[27] Niu F, Ré C, Doan A, Shavlik J (2011) Tuffy: Scaling up statistical inference in Markov logic networks using an RDBMS. Proc. VLDB Endow. 4:373–384.

[28] Suciu D, Olteanu D, Ré C, Koch C (2011) Probabilistic databases, synthesis lectures on data management. Morgan & Claypool.180 p.

[29] Wainwright MJ, Jordan MI (2008) Graphical models, exponential families, and variational inference. Found. Trends Mach. Learn. 1:1–305.

[30] Callison-Burch C, Dredze M (2010) Creating speech and language data with amazon's mechanical turk. In: Proceedings of the NAACL HLT 2010 workshop on creating speech and language data with Amazon's mechanical Turk. CSLDAMT ' 10:1–12.

[31] Mintz M, Bills S, Snow R, Jurafsky D (2009) Distant supervision for relation extraction without labeled data. In Proceedings of the Joint Conference of the 47th Annual Meeting of the ACL and the 4th International Joint Conference on Natural Language Processing of the AFNLP 2, ACL ' 09:1003–1011.

[32] Hoffmann R, Zhang C, Weld DS (2010) Learning 5000 relational extractors. In Proceedings of the 48th Annual Meeting of the Association for Computational Linguistics, ACL ' 10:286–295.

[33] Köpcke H, Thor A, Rahm E (2010) Evaluation of entity resolution approaches on real-world match problems. Proc. VLDB Endow. 3:484–493.

[34] Foote M (2000) Origination and extinction components of taxonomic diversity: general problems. Paleobiology 26:796–796.

[35] Miller AI, Foote M (1996) Calibrating the Ordovician radiation of marine life: implications for Phanerozoic diversity trends. Paleobiology 22:304–309. 11539204 10.1666/0094-8373-22.2.304

[36] Alroy J (2010) Geographical, environmental and intrinsic biotic controls on Phanerozoic marine diversification. Palaeontology 53:1211–1235.

[37] Sepkoski JJ Jr (1993) 10 years in the library: new data confirm paleontological patterns. Paleobiology 19:43–51. 11538041 10.1017/s0094837300012306

[38] Adrain JM, Westrop SR (2000) An empirical assessment of taxic paleobiology. Science 289:110–112. 10884223 10.1126/science.289.5476.110

[39] Ausich WI, Peters SE (2005) A revised macroevolutionary history for Ordovician–Early Silurian crinoids. Paleobiology 31:538–551.

[40] Niu F, Zhang C, Ré C, Shavlik JW (2012) DeepDive: web-scale knowledge-base construction using statistical learning and inference. VLDS 12:25–28.

[41] Van Noorden R (2014) Elsevier opens its papers to text-mining. Nature 506:17–17. 24499898 10.1038/506017a

[42] Smith AB (2001) Large-scale heterogeneity of the fossil record: implications for Phanerozoic biodiversity studies. P Trans R Soc B 356:351–367. 10.1098/rstb.2000.0768 PMC1088432 11316484

[43] Peters SE, Foote M (2001) Biodiversity in the Phanerozoic: a reinterpretation. Paleobiology 27:583–601.

[44] Patterson C, Smith AB (1989) Periodicity in extinction: the role of systematics. Ecology 70:802–811.

[45] Dubois A (2007) Naming taxa from cladograms: a cautionary tale. Mol Phyl Evol 42(2):317–330. 10.1016/j.ympev.2006.06.007 16949307

[46] Laurin M (2010) The subjective nature of Linnaean categories and its impact in evolutionary biology and biodiversity studies. Contrib Zool 79(4):131–146.

[47] Payne JL, Boyer AG, Brown JH, Finnegan S, Kowalewski M, et al (2009) Two-phase increase in the maximum size of life over 3.5 billion years reflects biological innovation and environmental opportunity. P Natl Acad Sci USA 106:24–27. 10.1073/pnas.0806314106 PMC2607246 19106296

[48] Laurin M (2004) The evolution of body size, Cope's rule and the origin of amniotes. Sys Biol 53(4):594–622. 10.1080/10635150490445706 15371249

[49] Finarelli JA, Flynn JJ (2006) Ancestral state reconstruction of biddy size in the Caniformia (Carnivora, Mammalia): the effects of incorporating data from the fossil record. Sys Biol 55(2):301–313. 10.1080/10635150500541698 16611601

[50] Slater GJ (2013) Phylogenetic evidence for a shift in the mode of mammalian body size evolution at the Cretaceous-Palaeogene boundary. Methods Ecol Evol 4(8):734–744.

