# Supplementary material for: A Machine Reading System for Assembling Synthetic Paleontological Databases
Source: PLoS One. 2014 Dec 1;9(12):e113523. doi: 10.1371/journal.pone.0113523 (PMC4250071; doi:10.1371/journal.pone.0113523)
Supplement: File S1 — Figure S1 in File S1 Schematic representation of the PDD workflow. Figure S2 in File S1 Overview of PDD feature extraction. Text, tables, and images in an original document are parsed (e.g., by table position extraction or natural language). Two or more entities and the specific properties in the document (i.e., features) that relate them are expressed as a row in a database. Figure S3 in File S1 Overview of factor graph component of PDD. Existing knowledge bases, such as data in the PBDB, are used to assess mention-level relations during distant supervision. Variables assessed for accuracy become evidence variables for statistical inference and learning steps. Figure S4 in File S1 Screen shot of web user interface used in blind experiment conducted by 7 human annotators. A unique link and instructions to complete the form were emailed to each participant, along with written instructions describing the nature of the task. Figure S5 in File S1 Summary of results of annotation experiment of PDD and PBDB taxonomic extractions. Human volunteers with varying levels of professional investment in the PDD were asked to assess a mixture of human- and machine-extracted data. Figure S6 in File S1 Summary of results of annotation experiment of occurrence data. Human volunteers were asked to assess human-generated occurrence data in the PDD. Although no indication was given as to the source of the data, many of the annotators assumed that they were evaluating machine-generated data. Figure S7 in File S1 PDD genus-level diversity (black curve) calculated using occurrences with period level or finer temporal resolution. The results in this figure can be compared to the epoch or finer temporal resolution used to generate the results in Fig. 2c. Figure S8 in File S1 Geographic distribution of occurrences in PDD-generated database. Geographic coordinates were extracted for each fossil occurrence extracted by PDD, primarily by identifying location entities linked to geological formati [file pone.0113523.s001.pdf]

SUPPLEMENTARY INFORMATION

A Machine Reading System for Assembling Synthetic Paleontological Databases

Shanan E. Peters [*], Ce Zhang [†], Miron Livny [†], and Christopher Ré [‡]

[*] Department of Geoscience, University of Wisconsin-Madison, Madison, WI, 53706 USA
[†] Department of Computer Science, University of Wisconsin-Madison, Madison, WI, 53706 USA
[‡] Department of Computer Science, Stanford University, Stanford, CA 94305 USA

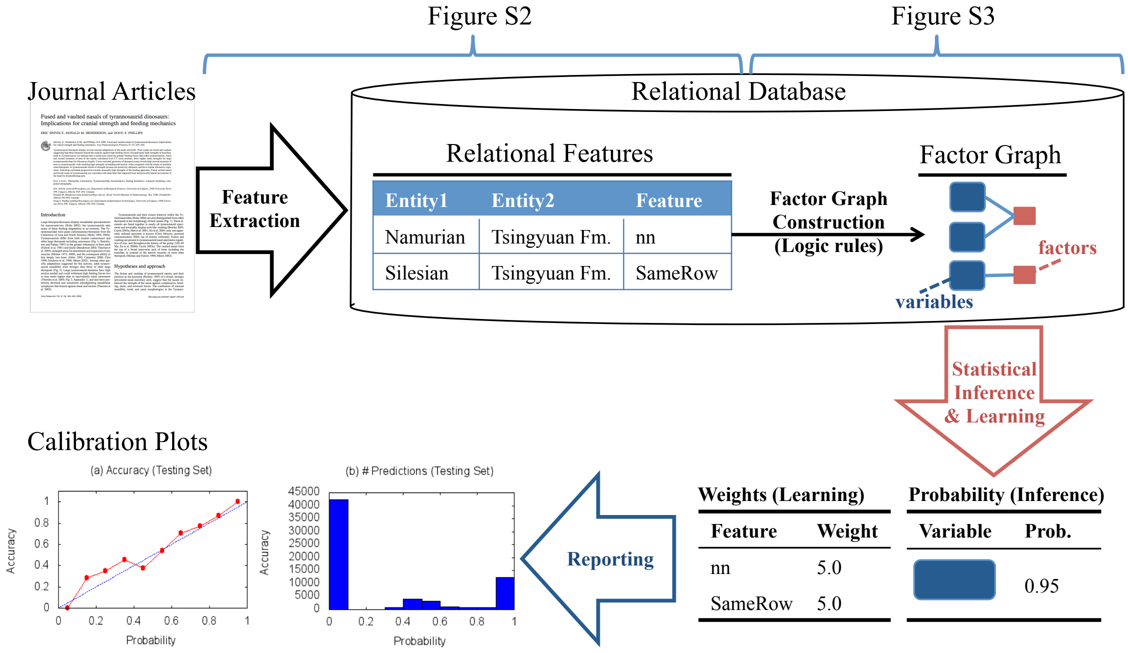

Supplementary Figure 1. Schematic representation of the PDD workflow.

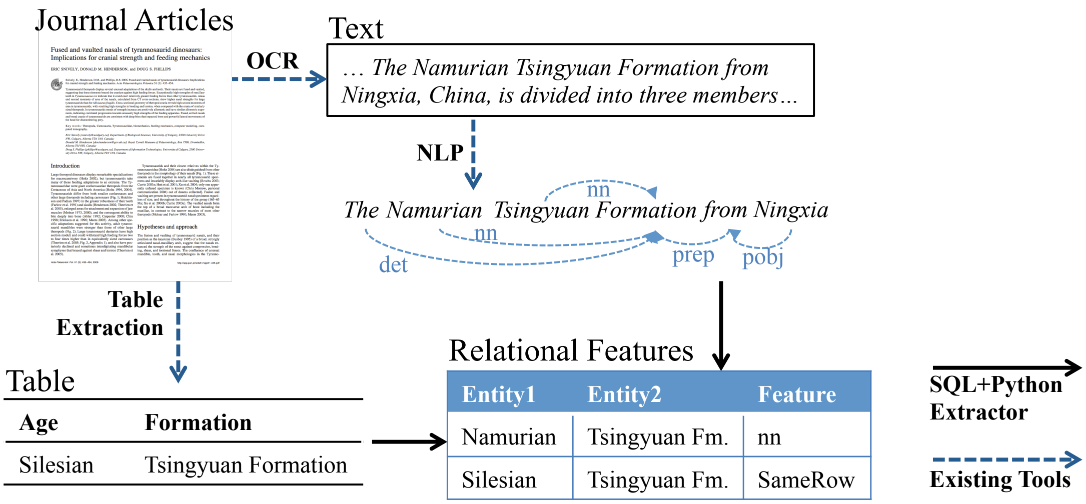

Supplementary Figure 2. Overview of PDD feature extraction. Text, tables, and images in an original document are parsed (e.g., by table position extraction or natural language). Two or more entities and the specific properties in the document (i.e., features) that relate them are expressed as a row in a database.

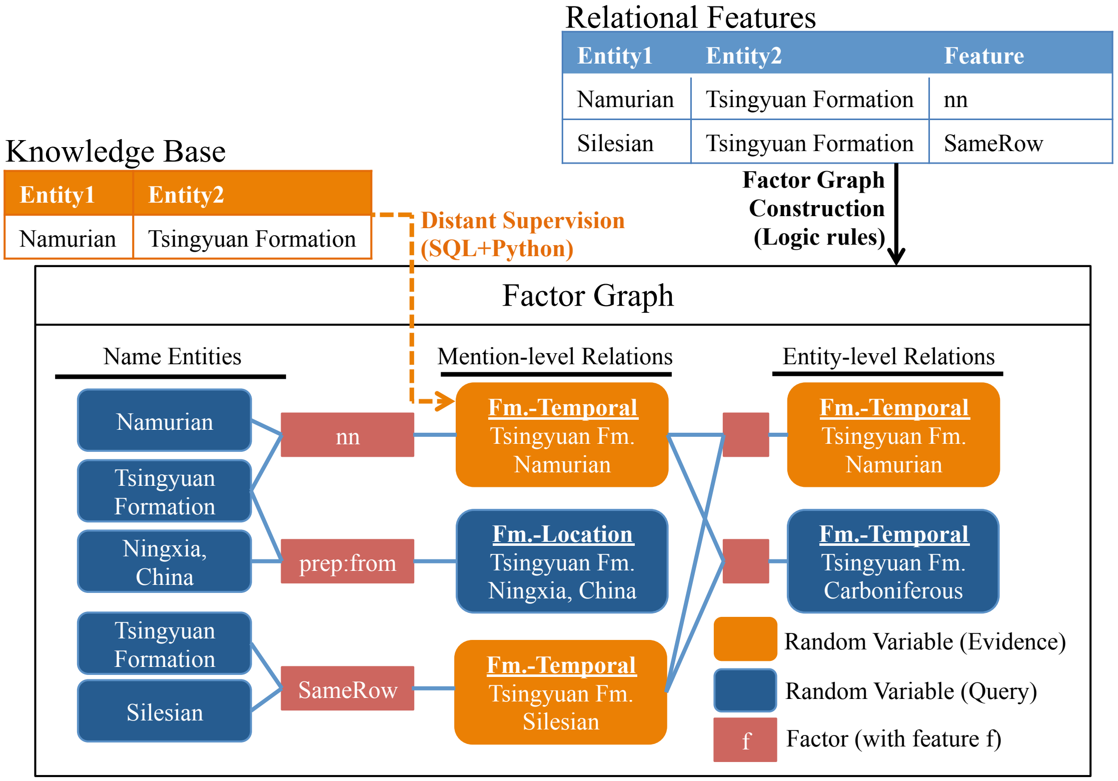

Supplementary Figure 3. Overview of factor graph component of PDD. Existing knowledge bases, such as data in the PBDB, are used to assess mention-level relations during distant supervision. Variables assessed for accuracy become evidence variables for statistical inference and learning steps.

taxon	rank	status	taxon	rank	in ref	not in ref	incorrect	?
Bellerophon carbonarius	species	belongs to	*Euphemites*	genus				
Bellerophon urii	species	belongs to	*Euphemites*	genus				
Bucaniopsis hibernicus	species	belongs to	*Retispira*	genus				
Euphemus konincki	species	belongs to	*Euphemites*	genus				

Supplementary Figure 4. Screen shot of web user interface used in blind experiment conducted by 7 human annotators. A unique link and instructions to complete the form were emailed to each participant. The wording of the instructions was as follows:

1. *"in ref" means you can find this *exact* fact in the document somewhere.*
2. *"not in ref" means you can't find the exact fact in the document anywhere (can include typos).*
3. *"incorrect" means it is an incorrect fact (e.g., wrong assignment/relationship, etc.).*
4. *"?" means you don't understand the fact in relation to document.*

Simply clicking on the box selects it for you. You can change it etc. as you go along. Once you are done, you can go to another ref by clicking on bottom. You can come back to the ref and inspect it to make sure it looks good, change things.

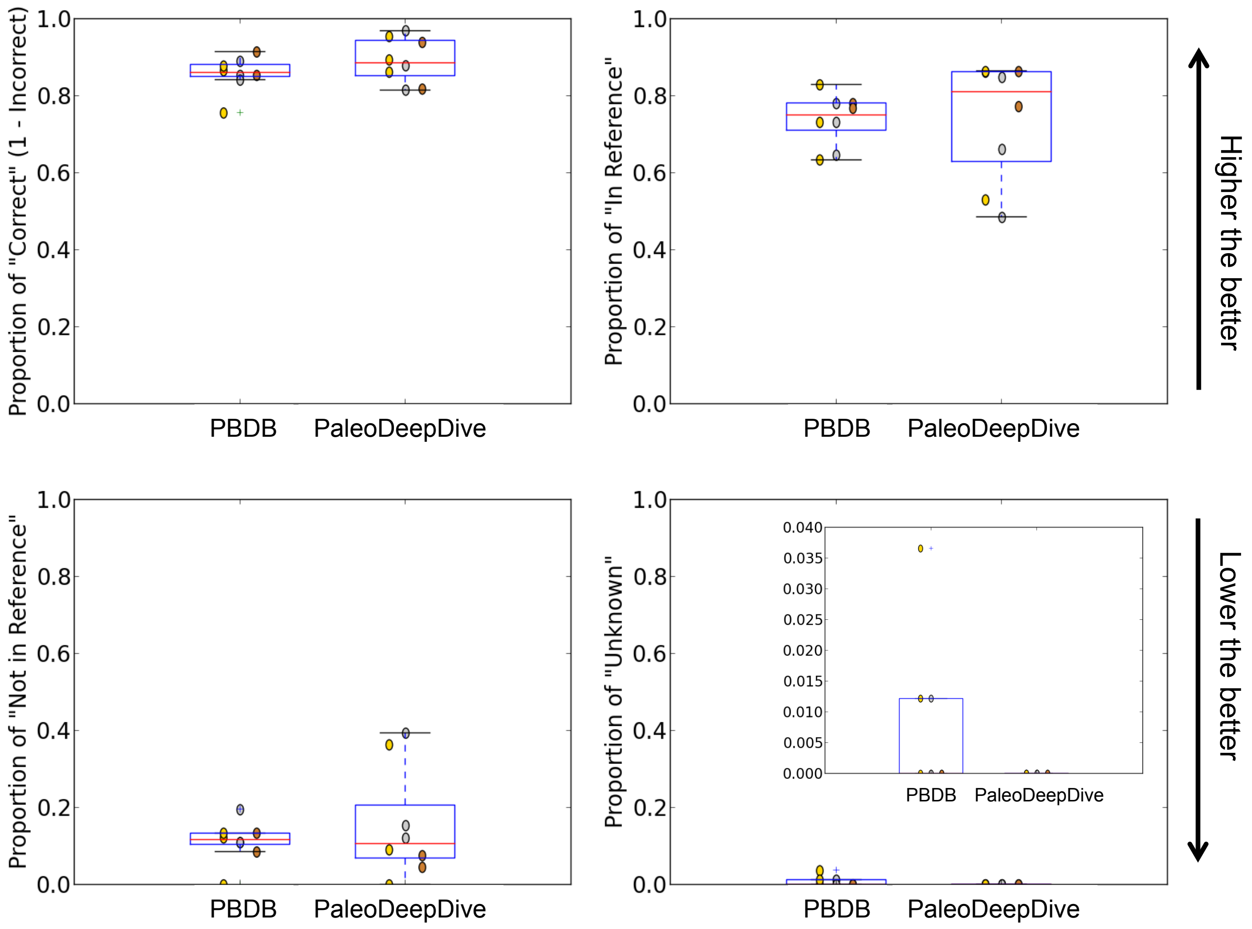

Supplementary Figure 5. Summary of results of annotation experiment of PDD and PBDB taxonomic extractions. Yellow, annotators with heavy PBDB governance involvement; blue, past governance involvement; red, graduate students.

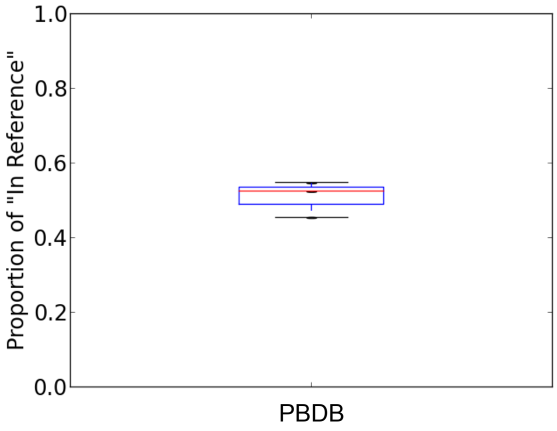

Supplementary Figure 6. Summary of results of annotation experiment of occurrence data, or (taxon, geologic unit, temporal interval) tuples in human-constructed PBDB. Results are for 3 volunteers, one from each of groups in Supplementary Figure 4.

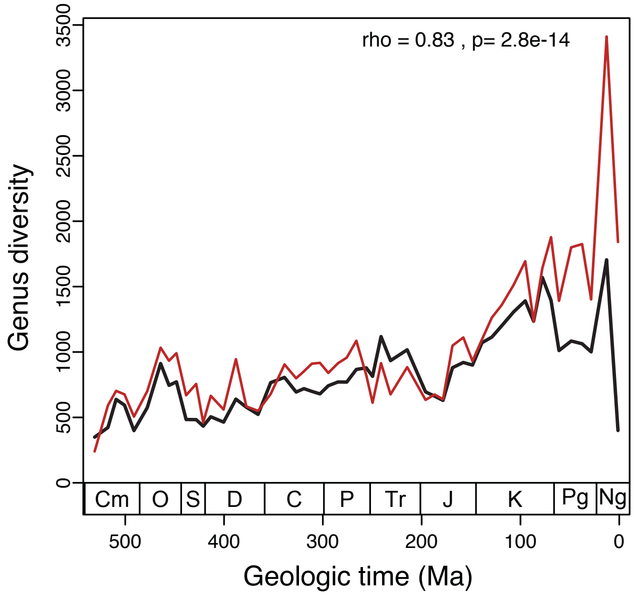

Supplementary Figure 7. PDD genus-level diversity (black curve) calculated using occurrences with period level or finer temporal resolution, as opposed to epoch or finer temporal resolution used in Fig. 1. The red curve shows PBDB data and is identical to the red curve in Fig. 1c.

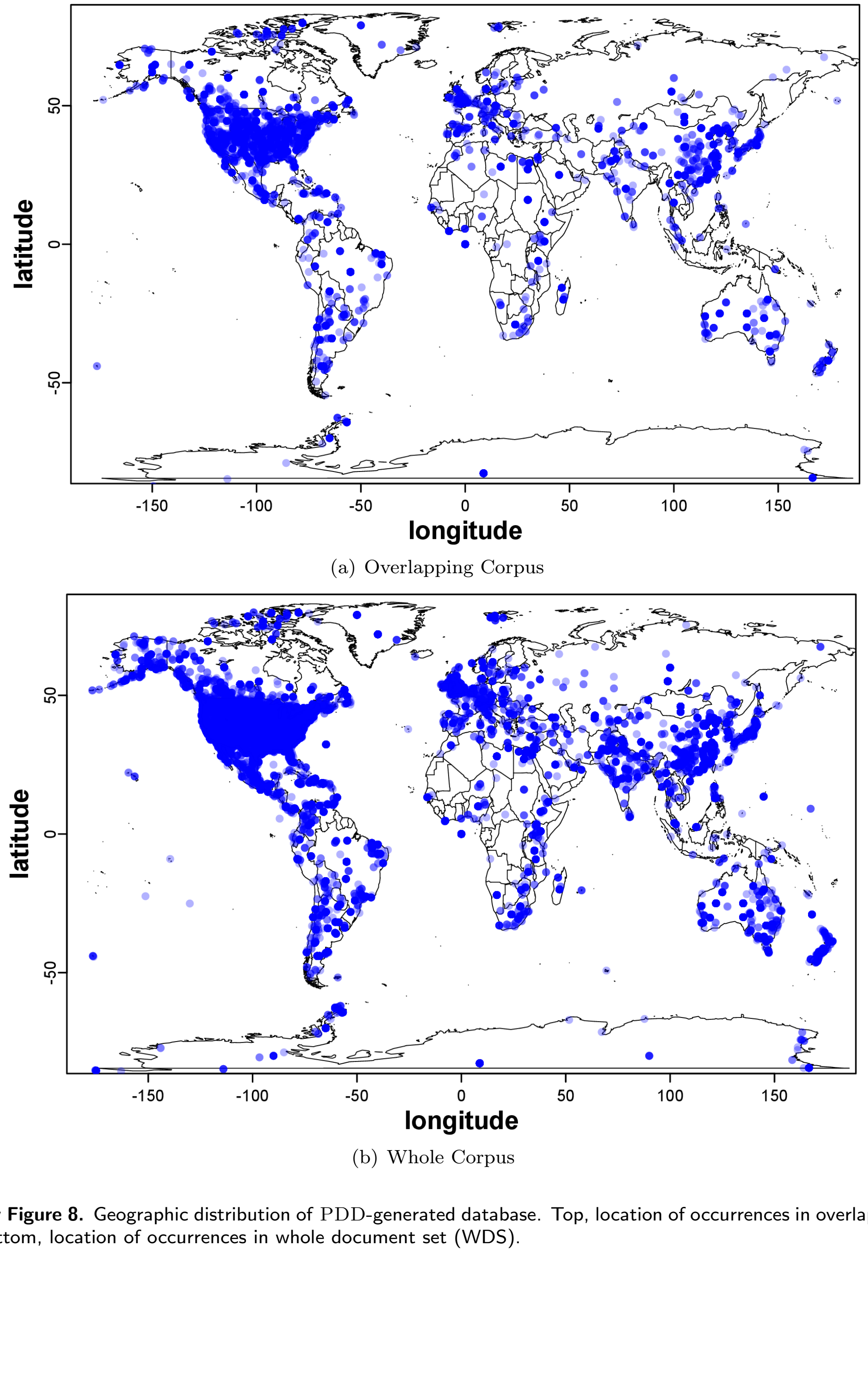

(a) Overlapping Corpus

(b) Whole Corpus

Supplementary Figure 8. Geographic distribution of PDD-generated database. Top, location of occurrences in overlapping document set (ODS). Bottom, location of occurrences in whole document set (WDS).

Layer	Features
Name Entities	Dictionary (English dictionary, GeoNames, PaleoDB, Species2000, Microstrat, MySQL stop words)<br>Part-of-speech tag from StanfordCoreNLP<br>Name-entity tag from StanfordCoreNLP<br>Name entity mentions in the same sentences (paragraphs, or documents)
Mention-level Relations	Word sequence between name entities<br>Dependency path between name entities<br>Name-entity tag from StanfordCoreNLP<br>Table caption-content association<br>Table cell-header association<br>Section headers (for Taxonomy)
Entity-level Relations	Temporal interval containment (e.g., Namurian $\subseteq$ Carboniferous)<br>Location containment (e.g., Ningxia, China $\subseteq$ China)<br>One formation does not likely span $>$ 200 million years

Supplementary Table 1. List of features and rules used in the current verison of PDD. Finding the right simple features and rules can be difficult. The PDD system is designed to operate in an iterative fashion, with error analysis occurring after each round of feature and rule definition.

Relation	Tuple in Knowledge	Positive Examples	Negative Examples
Taxonomy	(Taxon, Taxon) (t_1, t_2)	$\{(t_1, t_2)\}$	$\{(t_1, t_2') : t_2' \neq t_2\}$
Formation	(Taxon, Formation) (t, f)	$\{(t, f)\}$	Positive examples of other relations
Formation-Temporal (Mention)	(Formation,Interval) (t, i)	$\{(t, i') : intersect(i, i')\}$	$\{(t, i') : \neg intersect(i, i')\}$
Formation-Temporal (Entity)	(Formation,Interval) (t, i)	$\{(t, i') : intersect(i, i') \wedge \neg contain(i', i)\}$	$\{(t, i') : \neg intersect(i, i')\}$
Formation-Location (Mention)	(Formation,Location) (t, l)	$\{(t, l') : intersect(l, l')\}$	$\{(t, l') : \neg intersect(l, l')\}$
Formation-Location (Entity)	(Formation,Location) (t, l)	$\{(t, l') : intersect(l, l') \wedge \neg contain(l', l)\}$	$\{(t, l') : \neg intersect(l, l')\}$

Supplementary Table 2. List of distant supervision rules used in PDD. Function $contain(x, y)$ and $intersect(x, y)$ return True if the interval (or locations) x contains or intersects with y.

Journal Name	PBDB	PDD Overlapping Set	Coverage
Journal of Paleontology	2,667	2,534	95%
Journal of Vertebrate Paleontology	1,909	1,292	68%
Palaeontology	879	748	85%
Paleontological Journal	849	0	0%
American Museum Novitates	513	433	84%
NULL	509	0	0%
Acta Palaeontologica Polonica	483	433	90%
Nature	452	340	75%
Cretaceous Research	424	421	99%
Gobios	423	296	70%
Ameghiniana	394	21	5%
Canadian Journal of Earth Sciences	336	281	84%
Palaeogeography, Palaeoclimatology, Palaeoecology	325	317	98%
Vertebrata PalAsiatica	322	203	63%
Science	309	184	60%
Bulletin of the American Museum of Natural History	293	214	73%
Geological Magazine	269	24	9%
Alcheringa	268	0	0%
American Journal of Science	257	53	21%
Palaeontologische Zeitschrift	241	0	0%
Journal of Mammalogy	234	147	63%
Acta Palaeontologica Sinica	232	3	1%
United States Geological Survey Professional Paper	231	156	68%
Zoological Journal of the Linnean Society	203	200	99%
Contributions from the Museum of Paleontology, University of Michigan	195	174	89%
Palaeontographica Abteilung A	194	0	0%
Facies	187	0	0%
Lethaia	183	178	97%
Quarterly Journal of the Geological Society of London	180	122	68%
Zootaxa	180	0	0%
Palaios	174	164	94%
Annals of Carnegie Museum	172	25	15%
Proceedings of the United States National Museum	149	0	0%
Neues Jahrbuch fr Geologie und Paleontologie, Abhandlungen	147	0	0%
Review of Palaeobotany and Palynology	147	146	99%
American Journal of Botany	147	87	59%
Proceedings of the Academy of Natural Sciences of Philadelphia	142	40	28%
Journal of Human Evolution	135	122	90%
Proceedings of the National Academy of Sciences	133	51	38%
Journal of Systematic Palaeontology	132	27	20%
Geodiversitas	131	0	0%
Acta Geologica Sinica	130	78	60%
Bulletins of American Paleontology	129	0	0%
Bulletin de la Societe Geologique de France	122	0	0%
Palontologische Zeitschrift	115	0	0%
Rivista Italiana di Paleontologia e Stratigrafia	115	0	0%
Psyche	111	1	1%
Annals of the South African Museum	104	0	0%
Tulane Studies in Geology and Paleontology	103	0	0%
Paleontological Research	102	92	90%
Other Sources	30,851	2,175	7%
**Total**	47,632	11,782	25%

Supplementary Table 3. Distribution of documents in the overlapping document set. "NULL" corresponds to a NULL title document type field in the PBDB.

Taxon Name	Rank	Not Found on Google (Error Candidate)
Cirquella espinata	species	
Echinophyllia orpheensis	species	
Fenestella huascatayana	species	
Epigondolella primitia	species	
Palaeospheniscus gracilis.	species	
Pygurus carinatus	species	×
Arionellus tripunctatus	species	
Phacostylus amphistylus	species	
Circotheca multisulcatus	species	
Aulotortus praegaschei	species	
Leptaena demissa	species	
Xinjiangchelys laticentralis	species	
Conotreta lanensis	species	×
Martellia ichangensis	species	
Procavia antiqua	species	
Chermidae	family	
Monophyllus cubanus	species	
Gazella soemmeringi	species	
Pinna subspatulata	species	
Polacanthus faxi	species	×
Homotherium latidens	species	
Platanus primaeva	species	
Rhopalocanium satelles	species	
Cryptobairdia forakerensis	species	
Naiadites elongata	species	
Staurocephalus murchisoni	species	
Serpula anguinus	species	
Glycymeris angusticostata	species	
Eomunidopsis eutecta	species	
Actinocrinites gibsoni	species	
Zhelestes tes	species	×
Spinocyrtia ascendens	species	
Belemnopsis alexandri	species	
Agaricocrinus nodulosus	species	
Oreochromis shiranus	species	
Atrichornithidae	family	
Neltneria jaqueti	species	
Eurydice affinis	species	
Nummulites burdi	species	
Diacalymene marginata	species	
Scapteriscus didactylus	species	
Enhydriodon campanii	species	
Offneria nicoli	species	×
Propetrosia pristina	species	
Podocarpus campbelli	species	
Graffhamicrinus aristatus	species	
Productina sampsoni	species	
Bufina bicornuta	species	
Coccolithus staurion	species	
Ernanodon vas	species	×

Supplementary Table 4. Error Analysis of Taxon Entity Extractions in PDD

Reference No.	Genus	Correct	Extracted by PBDB
28945	Acrodenta	✓	
	Mastodonsaurus	✓	
	Mesodapedon	✓	
	Rhynchosaurus	✓	✓
	Scaphonyx	✓	
	Spirorbis	✓	
	Stenaulorhynchus	✓	
34109			
28146			
38697	Hazelia	✓	✓
	Leptomitus	✓	✓
32675			
33994	Gastropoda		
	Heterostropha	✓	
	Mathilda	✓	
	Mollusca	✓	
	Stenoglossa	✓	
27115			
41374	Archaeopterodactyloidea	✓	
	Beipiaopterus	✓	
	Boreopteridae	✓	
	Boreopterus	✓	
	Eopteranodon	✓	
	Eosipterus	✓	
	Feilongus	✓	
	Gegepterus	✓	
	Moganopterus	✓	✓
	Ningchengopterus	✓	
	Ornithocheiroidea	✓	
	Zhenyuanopterus	✓	
12054			
13061	Bactrosaurus	✓	
	Dyoplosaurus	✓	
	Gorgosaurus	✓	
	Hypacrosaurus	✓	
	Mandschurosaurus	✓	✓
	Nodosauridae	✓	✓
	Tanius	✓	
Human Recall			18%

Supplementary Table 5. Error Analysis: PDD Extractions

Reference No.	Genus	Correct	Extracted by PDD	Error Reason
28945	Rhynchosaurus	✓	✓	
34109	Austromola	✓		Not enough context features
	Odontoceti	✓		Not enough context features
28146	Cerapoda	✓		Not enough context features
38697	Hazelia	✓	✓	
	Leptomitus	✓	✓	
	Protospongia	✓		Not enough context features
32675	Tommotia	✓		Not enough context features
33994	Anticonulus	✓		Table recognition failure
	Ataphrus	✓		
	Austriacopsis	✓		
	Discohelix	✓		
	Emarginula	✓		
	Eucyclidae	✓		
	Eucyclus	✓		
	Guidonia	✓		
	Neritopsis	✓		
	Plectotrochus	✓		
	Proacirsa	✓		
	Pseudorhytidopilus	✓		
27115	Astreptodictya	✓		OCR error
	Athrophragma	✓		
	Batostoma	✓		
	Bryozoa	✓		
	Bythopora	✓		
	Calopora	✓		
	Coeloclema	✓		
	Constellaria	✓		
	Contexta	✓		
	Diploclema	✓		
	Echinodermata	✓		
	Graptodictya	✓		
	Helopora	✓		
	Nicholsonella	✓		
	Ottoseetaxis	✓		
	Pachydictya	✓		
	Phylloporina	✓		
	Porifera	✓		
	Prasopora	✓		
	Spongiostroma	✓		
	Stictopora	✓		
	Stictoporella	✓		
	Trilobita	✓		
41374	Moganopterus	✓	✓	
12054	Neosaurus	✓		Not enough context features
13061	Mandschurosaurus	✓	✓	
	Nodosauridae	✓	✓	
PDD Recall			11%	

Supplementary Table 6. Error Analysis: PBDB Extractions

**Relation**	**PBDB**	**PDD**	$p = 0.05$
Taxonomy	92%	97%	0
Temporal	89%	96%	+
Location	90%	92%	0
Formation	84%	94%	+

Supplementary Table 7. Comparison of Accuracies of PDD and PBDB. The column $p = 0.05$ is the significant test of one-tail Welch's t-test, where "+" means significant given the corresponding p-value, and "0" otherwise. The value 0.05 is picked by following the default setting of R.

Journal Name	1845- -1959	1960- -1969	1970- -1979	1980- -1989	1990- -1999	2000- -2009	2010 -2013	Total
American Journal of Science	2489		727	41		245	138	3640
American Midland Naturalist	2893	1022	1149	989	852	842	189	7936
American Museum Novitates	1974	413	288	272	320	388	98	3753
Annales de Palontologie					29	206	73	308
Annals of Carnegie Museum						82	38	120
Bulletin of the American Museum of Natural History	1318	93	105	72	52	196	65	1901
Comptes Rendus Palevol						679	270	949
Cretaceous Research				287	457	732	393	1869
Geological Journal	136	418	338	1116	680	662	423	3773
Geological Society America Bulletin	276	796		788	1158	1089	486	4593
Geology			1177	2675	2990	3024	1261	11127
Global and Planetary Change				20	469	1070	376	1935
Gobios		13	442	1072	1294	753	167	3741
International Geology Review	87	1482	1780	1541	724	635	353	6602
Journal of Asian Earth Sciences					149	1162	1123	2434
Journal of Geology	5782	736	929	754	671	516	153	9541
Journal of Human Evolution			859	890	759	1067	597	4172
Journal of Mammalogy	3023	1633	1509	1452	1336	1506	438	10897
Journal of Paleontology	2552	1500	1438	1297	1172	2224	643	10826
Journal of South American Earth Sciences				79	423	666	414	1582
Journal of Systematic Palaeontology						113	110	223
Journal of Vertebrate Paleontology				365	636	2152	934	4087
Journal of the Geological Society					329	946	346	1621
Lethaia		104	830	978	992	738	371	4013
Mammalian Species		1	122	224	284	216		847
Marine Micropaleontology			85	262	469	646	156	1618
Micropaleontology	202	375	302	264	270	316		1729
New Zealand Journal of Geology and Geophysics	121	733	730	519	484	403	115	3105
PALAIOS				290	567	677	237	1771
Palaeogeography, Palaeoclimatology, Palaeoecology		191	600	1108	1812	3221	1191	8123
Palaeontology	48	461	477	446	493	1470	560	3955
Palaios						620	287	907
Paleobiology			184	422	337	866	260	2069
Paleontological Research						192	88	280
Palynology			45	140	132	232	119	668
Proc. of AASP			79					79
Proceedings of the Geologists' Association	3514	430	415	416	404	394	273	5846
Quarterly Journal of the Geological Society of London	3063	177	19					3259
Review of Palaeobotany and Palynology		241	427	705	1031	887	406	3697
Revue de Micropaleontologie					104	262	72	438
Rocky			88	118	77	96	33	412
The Micropaleontologist	163							163
Transactions of the Kansas Academy of Science	2107	611	307	263	236	293	48	3865
USGS Open-File Report	403	466	2399	6480	5060	726	243	15777
United States Geological Survey Bulletin	2302	626	320	614	454	1	1	4318
United States Geological Survey Professional Paper	596	721	733	465	227	71	54	2867
Zoological Journal of the Linnean Society	1165	121	363	483	487	638	392	3649
Acta Palaeontologica Polonica	50	118	180	196	242	564	272	1622
Canadian Journal of Earth Sciences		530	1865	1981	1643	1077	377	7473
Oklahoma Geology Notes		15	58	60	56	39	3	231
Vertebrata Palasiatica	136	237	225	333	262	272	119	1584
Biodiversity Heritage Library								97129
**Total**								**277309**

Supplementary Table 8. Statistics of Whole Document Set (WDS).

	ODS	WDS	Ratio (WDS/ODS)
# Variables	13,138,987	292,314,985	22×
# Evidence Variables	980,023	2,066,272	2×
# Factors	15,694,556	308,943,168	20×
# Distinct Features (Weight)	945,117	12,393,865	13×
Documents	11,782	280,280	23×

Supplementary Table 9. Factor graph statistics in the overlapping and whole document sets. Evidence variables are those variables for which distant supervision has contributed an expectation. The scaling of evidence variables from the ODS to the WDS reflects the fact that most of the training data used by PDD derives from the PBDB data in the ODS.

Year	Volume	Issue	Reference Title
1993	13	3, suppl.	Ontogenetic changes in hind limb proportions within the Ghost Ranch population of Coelophysis bauri
2003	23	3	New dromomerycids (Mammalia: Artiodactyla) from the middle Miocene Sharktooth Hill Bonebed, California, and the systematics of the cranioceratinins
2002	22	3	Paleontology and stratigraphy of the Tecolotlan Basin, Jalisco, Mexico
2004	24	3	A new Miocene sperm whale (Cetacea, Physeteridae) from Virginia
2003	23	3, suppl.	A preliminary Prosauropoda phylogeny with comments on Brazilian basal Sauropodomorpha
2005	25	3	A revised faunal list for the Carmel Church Quarry, Caroline County, Virginia
1994	14	3, suppl.	Preliminary report on the microvertebrate fauna from the Late Cretaceous Bauru strata near Peirpolis, Minas Gerais, Brazil
1993	13	3, suppl.	Sedminentology and taphonomy of the Little Houston Quarry, Morrison Formation (Upper Jurassic), northeast Wyoming
1986	6	3	
2002	22	3	A flora and faunal list of specimens recovered from the Big Pig Dig, Badlands National Park, South Dakota

Supplementary Table 10. A Random Sample of PBDB References in *Journal of Vertebrate Paleontology* that Do Not Appear in the Overlapping Corpus.

Year	Volume	Issue	Reference Title
1905	22	NULL	
2006	312	NULL	Comment on “The brain of LB1, Homo floresiensis”
1984	224	NULL	
1885	5	116	Lesquereux’s Cretaceous and Tertiary Flora
1991	251	NULL	New fossil evidence on the sister-group of mammals and early Mesozoic faunal distributions
1990	249	NULL	
1900	11	282	The vertebral formula in Diplodocus Marsh
1997	278	NULL	A tribosphenic mammal from the Mesozoic of Australia
1905	22	568	The occurrence of ichthyosaur-like remains in the Upper Cretaceous of Wyoimng
1934	79	2039	A change of names

Supplementary Table 11. A Random Sample of PBDB References in *Science* that Do Not Appear in the Overlapping Corpus.

		ODS	WDS	Ratio (WDS/ODS)
Mention-level Candidates	Taxon	6,049,257	133,236,518	22×
	Formation	523,143	23,250,673	44×
	Interval	1,009,208	16,222,767	16×
	Location	1,096,079	76,688,898	76×
	Opinions	1,868,195	27,741,202	15×
	Taxon-Formation	545,628	4,332,132	8×
	Formation-Temporal	208,821	3,049,749	14×
	Formation-Location	239,014	5,577,546	23×
Entity-level Result	Authorities	163,595	1,710,652	10×
	Opinions	192,365	6,605,921	34×
	Collections	23,368	125,118	5×
	Occurrences	93,445	539,382	6×
	Documents	11,782	280,280	23×

Supplementary Table 12. Extraction statistics for the overlapping and whole document sets. Authorities refers to distinct taxa (identified by name and, optionally, ranks and authors).

Relation	# Annotations	Precision	Recall
Taxonomy	933	97%	39%
Temporal	478	96%	69%
Location	655	92%	36%
Formation	2,271	94%	21%

Supplementary Table 13. Statistics of Annotations Collected and Quality Score for Each Relation

1 Extensions

1.1 Body Size Extraction

In order to extract body size estimates from biological illustrations, we need to extract the relation:

$$(Taxon, FigureName, FigureLabel, Magnification, ImageArea)$$

where $ImageArea$ is a region on the PDF with known DPI so that the actual size of the image on a printed document is known. The following table is an example of the target extracted relation.

Vediproductus wedberensis	Fig. 381	2a	X1	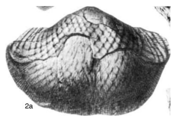
Compressoproductus compressus	Fig. 382	1a	X0.8	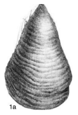
Devonoproductus walcotti	Fig. 383	1b	X2.0	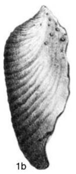

There were two steps in the process: (1) Image processing, and (2) text extraction. In PDD, these two components are done jointly in the same factor graph.

Image Processing. The goal of the image processing component is to associate each image area with a figure label. To achieve this, PDD needs to (1) detect image areas and figure labels from PDF documents, and (2) associate image areas with figure labels. Supplementary Figure 9 illustrates these two steps.

Detection of Image Areas and Figure Labels. The following steps were taken: (1) Edge detection; (2) Watershed Segmentation; (3) Image Dilation; and (4) Connected-component Detection (Supplementary Figure 9). Standard online-tutorials were followed, with one variant for Image Dilation. In this step, one needs to specify a parameter for dilation. Instead of specifying one value for the parameter, we tried a range of parameters and generate different versions of segmentations. PDD then trained a logistic regression classifier to choose between these segments trained on a human-labeled corpus.

Association of Image Areas with Figure Labels. After recognizing a set of image regions and their corresponding OCR results, PDD attempted to predict the association of figure labels and image areas, as shown in Supplementary Figure 9. Similar to relation extraction, PDD introduces a Boolean random variable for each label and image area pair. It then builds a logistic regression model using features such as the distance between label and image areas, and whether a label is nearest to an image area and vice versa.

Text Extraction. PDD also extracts information from text, as shown in Supplementary Figure 10. This extraction phase is similar to what was used when extracting fossil occurrence-related relations. In the name entity recognition component, PDD extracts different types of mentions, including Figure name (e.g., "Fig. 3"), Figure labels (e.g., "3a-c"), Taxon (e.g., "*B. rara*"), and magnitude (e.g., "X1"). Supplementary Figure 10 shows an example of these mentions (raw text with OCR errors). PDD then extracts relations between these mentions using the same set of features as other diversity-related relations.

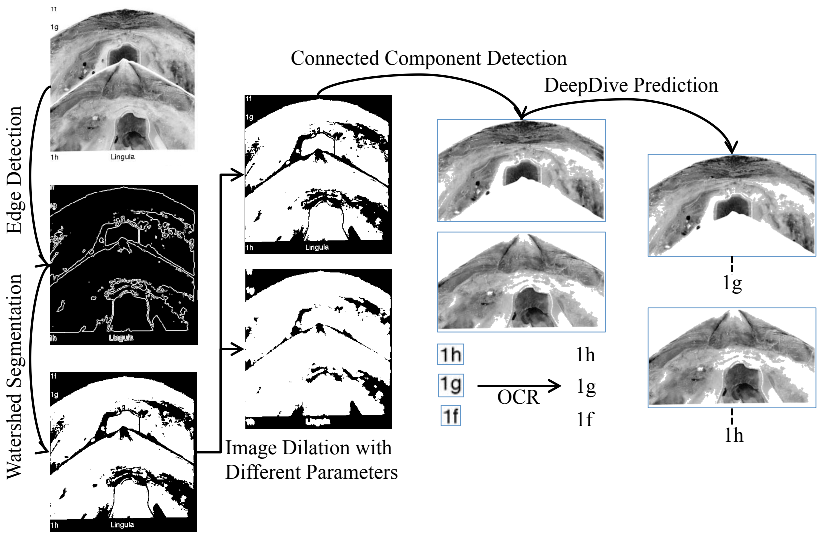

Supplementary Figure 9. Image Processing Component for Body Size Extraction. Note that this examples contains the illustration of a *partial* body.

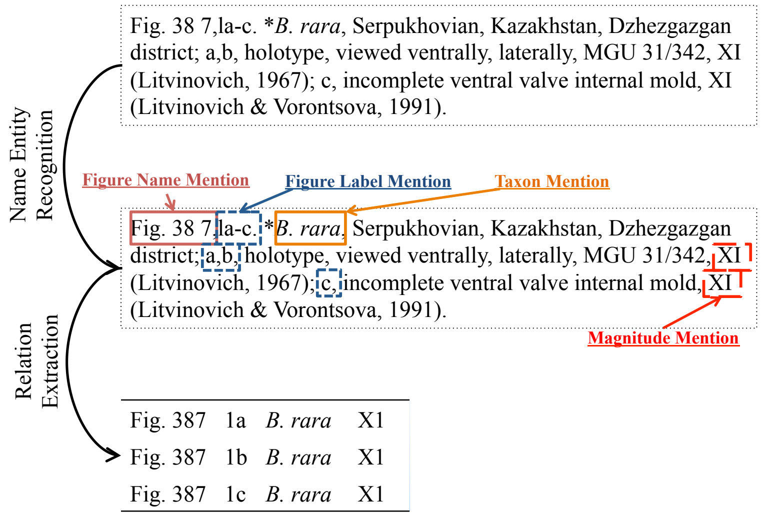

Supplementary Figure 10. Relation Extraction Component for Body Size Extraction.

Joint Inference. Both the image processing component and the text extraction component results in a factor graph populating two relations with schema

$$(FigureLabel, ImageArea)$$

and

$$(Taxon, FigureName, FigureLabel, Magnitude).$$

PDD joins these two intermediate relations to form a large factor graph to populate the target relation. Joint inference on the whole factor graph is then executed.

1.2 Body Size Extraction Validation

Corpus. Other researchers [1] recently compiled body size measurements by manually measuring illustrations and reading captions in the *Treatise on Invertebrate Paleontology.* Of the 55 volumes now accessible, humans have made measurements from part H, I, K, L, N, O, P, Q, R, S, T, U. We created from these documents the following three sets:

1. **Testing Corpus (With Ground Truth).** Part H.
2. **Testing Corpus (Without Ground Truth).** Part A, B, C, D, E, F, G, W, V.
3. **Training Corpus.** Part I, K, L, N, O, P, Q, R, S, T, U.

We used the Training Corpus to generate training data for distant supervision. We compared our results with those of human annotators using the Testing Corpus (With Ground Truth). The Testing Corpus (Without Ground Truth) shows that PDD helps to extend the body size database with new extractions that are not provided by human annotators.

Results on Testing Corpus (With Ground Truth). PDD is able to to achieve high precision and slightly higher recall than human when extracting body size measurements and their relations.

Precision. We measured the precision of PDD by randomly sampling 100 extracted instances of the target relation and manually annotate those extractions. We find that the accuracy is more than 92%.

Recall. We next counted the number of distinct (genus, figure name, figure label) tuples that are extracted by humans and PDD on the same set of documents. We find that human extracted 4,837 distinct tuples, and PDD extracted 5,783 distinct tuples, or 20% more. The primary reason for the increase is the complete extraction of meaurements for all parts of a figure (e.g., "1a-f"). Humans typically extract only one part.

Although selective data extraction is often a decision made for the sake of expediency and because not all images provide optimal orientations for the dimensions being targeted by a given investigation, extracting complete measurements and associated textual descriptions establishes the foundation for more complete morphometric analyses.

Results on Testing Corpus (Without Ground Truth). PDD is able to extract facts on documents that have not yet been processed by humans. PDD processed Parts A, B, C, D, E, F, G, V, W of the *Treatise on Invertebrate Paleontology*, which have not yet been processed for body size by [1]. PDD extracts 7K distinct (genus, figure name, figure label) tuples from these documents.

1.3 Multi-linguistic Extraction

Corpus. We followed a similar protocol as we used to collect the overlapping corpus for English documents. We identified the top-20 journals ranked by the number of journal articles in PBDB, and attempted to download articles from their web site. Access was limited to *Vertebrata Palasiatica* (Chinese), *Stuttgarter Beitrage zur Naturkunde* (German), and *Eclogae Geologicae Helvetiae* (German). A total of 1,583 Chinese journal articles and 4,393 German journal articles were obtained in this way. We used the same protocol to map these journal articles to articles in PBDB. Of these, there were 47 articles in Chinese and 56 German articles that overlapped with the PBDB.

	English	Chinese	German	Dictionary Source
Rock Formation	Formation	组	Formation	Manual
	Clay	石	Ton	
Temporal Interval	Late Cretaceous	晚白垩世	Oberkreide	Manual
	Cretaceous	白垩世	Kreide	
Location	United States	美国	Vereinigte Staaten	geonames.org
Taxon	*Aeschnidium densum*	*Aeschnidium densum*	*Aeschnidium densum*	All in Latin

Protocol. We compared the extractions of PDD in the overlapping set with the PBDB extractions on the same set of documents. Our way of assessing quality is recall for the tuple

$$(Taxon, TimeInterval)$$

This tuple is language-independent because (1) taxon has unified Latin-representation in all English, Chinese, and German articles; and (2) time Intervals and their hierarchical relationships are known by PDD for all languages. To extract this tuple, PDD requires the information in all other tuples, including $(Taxon, Formation)$, $(Formation, TimeInterval)$, and $(Formation, Location)$. We selected taxa common to both PDD and PBDB, and label PDD's extraction as correct if the taxon temporal ranges overlap.

Recall. From the overlapping corpus, PBDB extracts $(Taxon, TimeInterval)$ tuples for 85 distinct genera in Chinese and 242 distinct genera in German. We find that PDD correctly extracts $(Taxon, TimeInterval)$ for 24 genera (28%) in Chinese and 82 (33%) genera in German. The difference between Chinese and German is caused primarily by OCR quality, even though we used commercial OCR tools for both. Chinese has lower OCR quality because of the large vocabulary in East-Asian languages.

Precision. Out of all 24 distinct genera in Chinese and 82 distinct genera in German articles, we find that all of them overlap with PBDB extractions in terms of their temporal interval, indicating high precision.

2 Specific Technical Validation

Here we describe DeepDive, the underlying system that powers PDD [2–7].

2.1 Probabilistic Framework

2.1.1 Related Work

Knowledge Base Construction (KBC) has been an area of intense study over the last decade [8–19]. Within this space, there are a number of approaches.

Rule-based Systems. The earliest KBC systems used pattern matching to extract relationships from text. The most well known example is the "Hearst Pattern" proposed by Hearst [20] in 1992. In her seminal work, Hearst observed that a large amount of hyponyms can be discovered by simple patterns, e.g., "X, such as Y". Hearst's technique forms the basis of many further techniques that attempt to extract high quality patterns from text. In industry, rule-based (pattern-matching-based) KBC systems, such as IBM's SystemT [8, 21], have been built to develop high quality patterns. These systems provide the user a (usually declarative) interface to specify a set of rules and patterns to derive relationships. These systems have achieved state-of-the-art quality after carefully engineering effort as shown by Li et al. [21].

Statistical Approaches. One limitation of rule-based systems is that the developer needs to ensure that all rules provided to the system are high precision rules. For the last decade, probabilistic (or machine learning) approaches have been proposed to allow the system select between a range of a priori features automatically. In these approaches, the extracted tuple is associated with a marginal probability that it is true (i.e., that it appears in the KB). DeepDive, Google's knowledge graph, and IBM's Watson are built on this approach. Within this space there are three styles of systems:

- **Classification-based Frameworks** Here, traditional classifiers assign each tuple a probability score, e.g., naïve Bayes classifier, and logistic regression classifier. For example, KnowItAll [12] and TextRunner [13, 14] uses naïve Bayes classifier, and CMUs NELL [16, 17] uses logistic regression. Large-scale systems typically use these types of approaches in sophisticated combinations, e.g., NELL or Watson.

- **Maximum a Posteriori (MAP)** Here, the probabilistic approach is used but the MAP or Most likely world (which do differ slightly) is selected. Notable examples include the YAGO system [15],which uses a PageRank-based approach to assign a confidence score. Other examples include the SOFIE [10] and Prospera [11], which use an approach based on constraint satisfication.

- **Graphical Model Approaches** The classification-based methods ignore the interaction among predictions, and there is a hypothesis that modeling these correlations yields higher quality systems more quickly. A generic graphical model has been used to model the probabilistic distribution among all possible extractions. For example, Poon et al. [19] used Markov logic networks (MLN) [22] for information extraction. Microsoft's StatisticalSnowBall/EntityCube [18] also uses an MLN-based approach. A key challenge with these systems is scalability. For example, Poon et al. was limited to 1.5K citations. Our relational database driven algorithms for MLN-based systems are dramatically more scalable [3].

2.1.2 Calibrated Probabilities

DeepDive takes a Bayesian probabilistic approach to KBC by treating OCR, NLP, image processing, and feature recognition as one joint probabilistic inference problem in which all predictions are modeled as a factor graph (Fig. S3). This probabilistic framework ensures all facts that are produced by DeepDive are associated with a marginal probability.[1] These marginal probabilities are meaningful in DeepDive (i.e., they should correspond to the actual probabilities of a fact beig correct), which provides a mehcanism for evaluation and an aid to improving the system.

Calibration. In DeepDive, *calibration plots* are used as a way to summarize the overall quality of the KBC results. Ideally, the probability associated with a given fact in DeepDive should equal the empirical probability that this fact is correct (i.e., an extraction with a probability 0.95 should be correct with a 95% of the time when inspected in the original source). Because DeepDive uses a joint probability model, any set of predictions can be assigned a marginal probability. Queries can then be against the model to help determine where a model needs improvement.

Supplementary Figure 11 and Supplementary Figure 12 show calibration plots for the ODS and the WDS presented in the main text. We will use Supplementary Figure 11(1) as an example, which is the target relation Taxonomy in the ODS. A calibration plot contains three components: (a) Accuracy, which measures the test-set accuracy of a prediction with a certain probability; (b) # Predictions (Testing Set), which measures the number of extractions in the test set with a certain probability; and (c) # Predictions (Whole Set), which measures the number of extractions in the whole set with certain probability. The difference between test set and whole set is that the former has training labels for each random variable. Results are summarized as histograms, and empirically we find that a bin of size of 0.1 is usually sufficient to understand the behavior of the system.

[1] Cox's theorem asserts (roughly) that if one uses numbers as degrees of belief, then one must either use probabilistic reasoning or risk contradictions in a reasoning system, i.e., probabilistic reasoning is the only sound system for reasoning in this manner [23].

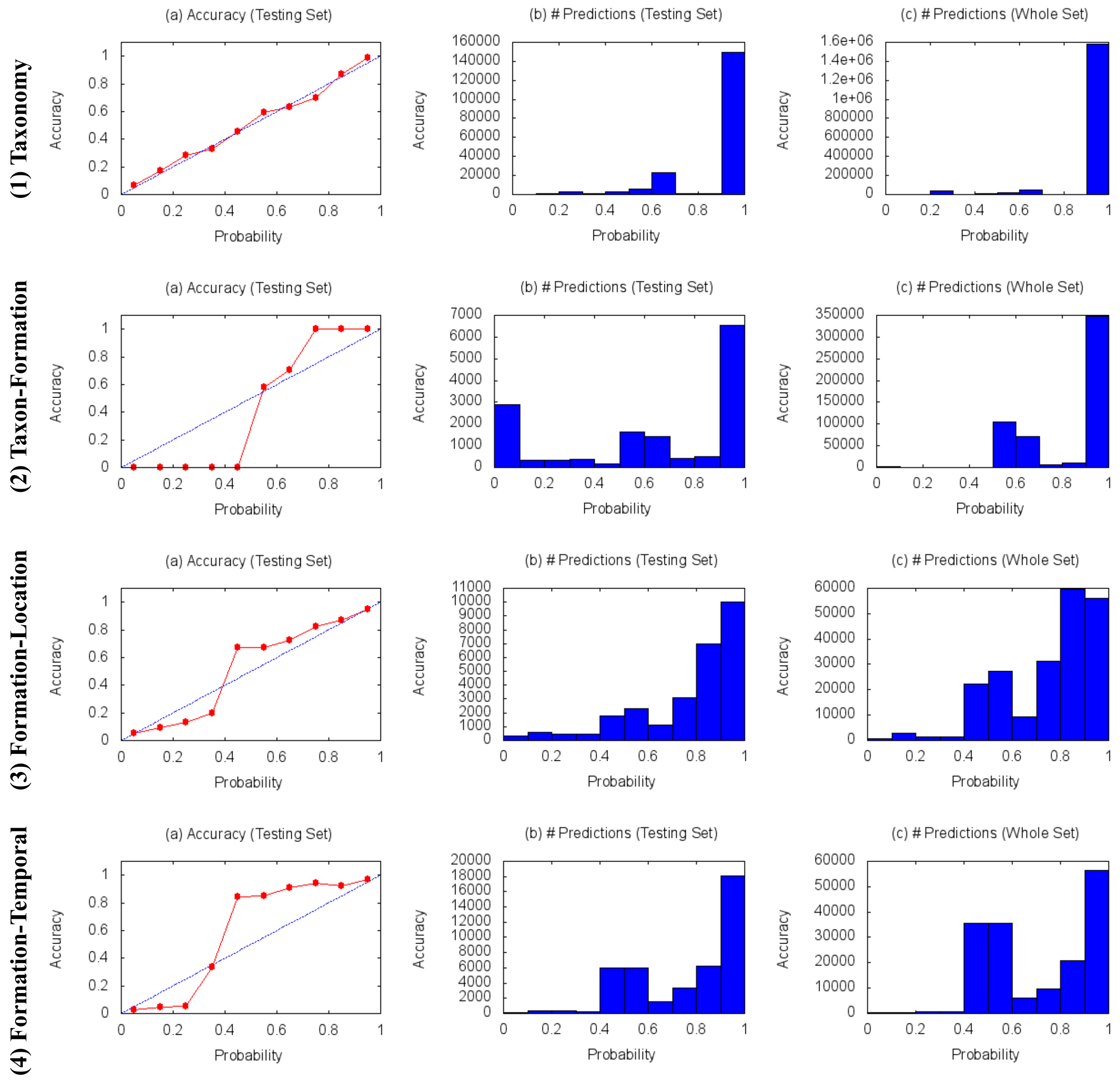

Supplementary Figure 11. Calibration Plots for All Relations on Overlapping Corpus

Using Calibration Plots

(a) Accuracy. If the accuracy curve is similar to the ideal (0,0)-(1,1) line, it means that a probability produced by the system matches the *test-set accuracy*. For example, Supplementary Figure 11(1) shows a reasonably good curve for calibration. Differnces in these two lines can be caused by (1) inefficient training data or a small testing corpus, and/or (2) bad mixing behavior of the sampler or other software bugs. For example, Supplementary Figure 12(2,3,4) shows a much better calibration behavior than Supplementary Figure 11(2,3,4), primarily because the former is based on the whole corpus, which has more training data and a larger testing set.

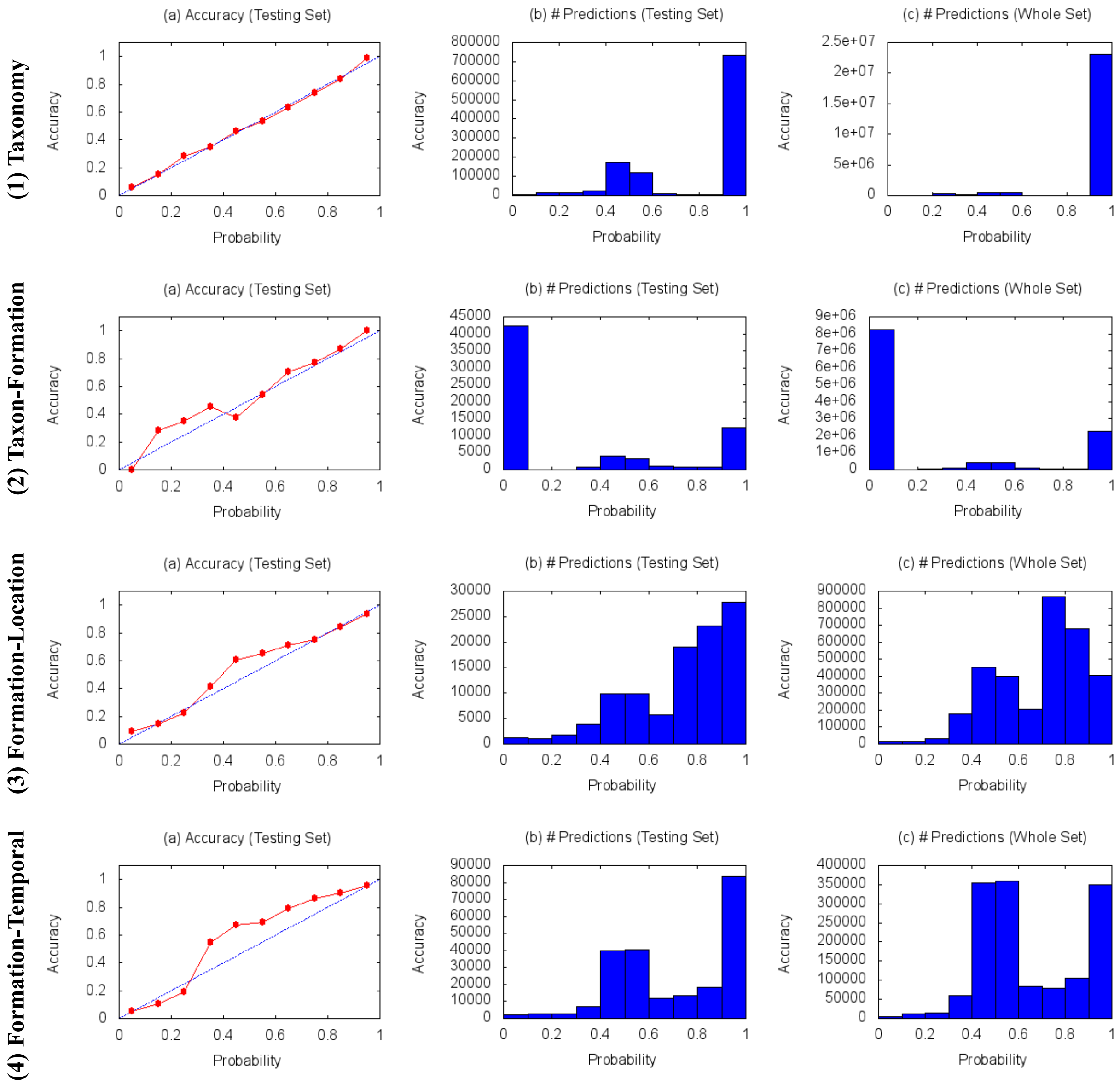

Supplementary Figure 12. Calibration Plots for All Relations on Whole Corpus

(b) # Predictions (Testing Set). Ideally, the # Predictions histogram should have a "U" shape. That is, most of the data are concentrate at high probability (where we are confident it is correct) and low probability (where we are confident it is incorrect). Large numbers of predictions with a probability approximately 0.5 means that the system has little information about how to classify these extractions. This implies that more features could be defined to resolve uncertainty. For example, Supplementary Figure 11(2) shows a U-shape curve with some masses around 0.5-0.6. The shape of the histogram relies on the ratio between the number of positive examples and negative examples. When the number of positive examples dominates negative examples and there is a bias term, it is possible that there are very small amount extractions with a probability near 0. Supplementary Figure 11(1,3,4) illustrate this phenomenon.

(c) # Predictions (Whole Set). This histogram is similar to (b), but illustrates the behavior of scaling the system to a set of documents for which we do not have any training examples. Usually we hope that (c) has a similar shape to (b).

Usage. The above techniques have proven critical to debugging and improving the quality of PDD. In response to low confidence, a user can provide labeled examples, which allows the system to learn weights that yield higher confidence. Additionally, a user may write logical inference rules that provide ways of improving quality, which is a key component of all statistical relational approaches.

2.2 Declarative Interface for Joint Inference and Rich Features

2.2.1 Related Work

Here we survey recent efforts that focus on how to improve the quality of a KBC system.

Rich Features. Different researchers have recently noted the importance of combining and using a rich set of features and signals to improve the quality of a KBC system. Two famous efforts, the Netflix challenge [24], and IBM's Watson [25], which won the Jeopardy gameshow, have identified the importance of features and signals:

> *Ferrucci et al. [25]: For the Jeopardy Challenge, we use more than 100 different techniques for analyzing natural language, identifying sources, finding and generating hypotheses, finding and scoring evidence, and merging and ranking hypotheses. What is far more important than any particular technique we use is how we combine them in DeepQA such that overlapping approaches can bring their strengths to bear and contribute to improvements in accuracy, confidence, or speed.*
>
> *Buskirk [24]: The top two teams beat the challenge by combining teams and their algorithms into more complex algorithms incorporating everybody's work. The more people joined, the more the resulting team's score would increase.*

In both efforts, the rich set of features and signals contributed to the high-quality of the corresponding system. Other researches have found similar phenomena. For example, Mintz et al. [26] finds that although both surface features and deep NLP features have similar quality for relation extraction tasks, combining them achieves a significant improvement over using either one in isolation. Similar "feature-based" approaches are also used in other domains (e.g., Finkel et al. [27] uses a diverse set of features to build a NLP parser with state-of-the-art quality). In our own work [28], we have also found that integrating a diverse set of deep NLP features can improve a table extraction system significantly.

Joint Inference. Another recent trend in building KBC system is to take advantage of *joint inference* [5, 19, 28–33]. Different from traditional models [34], such as logistic regression or SVM, joint inference approaches emphasize learning multiple targets simultaneously. For example, Poon et al. [19, 31] find that learning segmentation and extraction in the same Markov logic network significantly improves the quality of information extraction. Similar observations have been made by Min et al. [29] and McCallum [30]. Our recent work also show the empirical improvement of joint inference on the diverse set of tasks, including relation extraction [5] and table extraction [28].

Deep Learning and Joint Inference. A recent emerging effort in the machine learning community is to build a fully-joint model for NLP tasks [32,33]. The goal is to build a single joint model from the lowest level (e.g., POS tagging) to the highest level (e.g., semantic role labeling). The PDD system is built in a similar spirit that attempts to build a joint model for low-level tasks (e.g., OCR), to high-level tasks (e.g., cross-document inference of relation extraction).

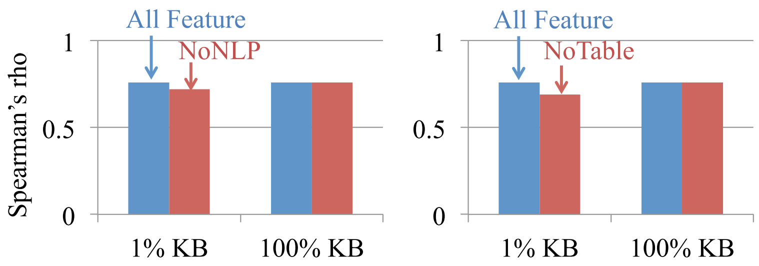

Supplementary Figure 13. Lesion Study of Deep NLP Features and Table Recognition

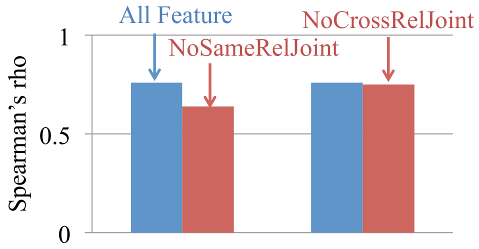

Supplementary Figure 14. Lesion Study of Joint Inference

2.2.2 The DeepDive Approach and the Impact of Rich Features and Joint Rules

DeepDive uses joint inference rules and rich features. In this section, we test that these features and rules are important to PDD's quality by conducting a lesion study.

Protocol. All experiments were run on the overlapping corpus as described in the main text. We produced variants of PDD by removing features/rules and all components that rely on the output of the removed feature/rule. We summarize the quality of PDD by computing Spearman's rho for first differences in genus-level biodiversity (as in Fig. 1).

Features. The PDD feature extraction phase extracts a set of features, including deep linguistic features, e.g., dependency parsing results, and vision-based features (e.g., a simple table extractor based on Hough Transform). To study their impact, we conduct lesion study by sequentiallydisabling these features.

Deep NLP Features. Supplementary Figure 13(a) shows the impact of removing NLP features (e.g., dependency path). If we use the whole PBDB is used, dropping these Deep NLP features does not have a significant effect on Spearman's rho. However, if the knowledge base used for training is reduced to 1% of it s size, then dropping NLP features results in a decrease of Spearman's rho from 0.72 from 0.82.

Vision-based Table Recognition. PDD contains a table recognition component to detect tables using vision-based features (e.g., Hough Transform). When disabling this component and using the 1% PBDB for distant supervision, PDD achieves a Spearman's rho of 0.69. This drop is the effect of decreased recall of data in tables.

Joint Inference Rules. PDD contains a set of factors for joint inference among random variables, as shown in Fig S3. We study their impact on two types of joint inference rules: (1) joint inference within one relation; and (2) joint inference across different relations (Supplementary Figure 14).

Joint Inference for Same Relations. Disabling all joint inference rules results in a Spearman's rho of 0.64, even when using the whole PBDB knowledge base. This is a marked decline from the Spearman's rho of 0.82 obtained when these rules are enabled. This large decline in quality is caused by the fact that jointly infering the values of random variable results in much higher-quality predictions. For example, assume that we have three candidate facts that Tsingyuan Formation has the age (1) Carboniferous ,(2) Namurian, and (3) Kungurian. In the current PDD system, the higher confidence for Carboniferous will also boost its confidence for Namurian (because of containment), and decrease its confidence for Kungurian (because Kungurian is so much younger than Carboniferous). This type of joint inference between random variables help PDD to produce result with higher recall (by boosting confidence to cross the imposed 0.95 threshold) and precision (by eliminating wrong predictions).

Joint Inference across Relations. The current PDD system has three joint inference rules across different relations (e.g., one geologic formation entity mention cannot be concurrently a location mention). We disable these rules and show in Supplementary Figure 14 that it does not have a large impact to the overall quality. This implies that the current PDD system is quite modular across different relations. This means that different types of relations can be decoupled and applied to other related applications (e.g., for biology or geology).

2.3 Scalability and High Performance Statistical Inference and Learning

2.3.1 Related Work

There is an emerging trend in both industry and academia to support statistical inference and learning, and we survey these efforts in this section.

Hardware Efficiency. One line of research tries to speed-up statistical inference and learning by better taking advantage of modern hardware and clusters. For example, many industrial database vendors have integrated statistical analytics components into their product. For example, Oracle's ORE [35], Pivotal's MADlib [36], and IBM's SystemML [37]. These systems provide functionalities like logistic regression and collapsed Gibbs sampling for topic modeling on their data management systems. There are also efforts to design new data processing framework instead of relying on the traditional database systems. Indeed, most data processing frameworks developed in the last few years are designed to support statistical analytics including Mahout [38] for Hadoop, MLI for Spark [39], GraphLab [40], GraphChi [41], and Delite [42, 43]. These systems have been shown to increase the performance of corresponding statistical analytics tasks significantly.

Statistical Efficiency. One key difference between statistical inference and learning with traditional SQL-like analytics is that different ways of executing the same tasks usually lead to different speed when converging to the same quality. Therefore, another line of related work, mainly contributed by the mathematical optimization and machine learning community, is to design more efficient algorithms for statistical inference tasks. One of the recent trends is to design lock-free algorithms that can be executed on the emerging multi-socket multi-core machines with high parallelism [3, 44–47]. For example, Tsitsiklis et al. [44] proves asymptotic convergence for a parallel coordinate descent algorithm, and Bradley et al. [47] proves the convergence rate and theoretical speedups for parallel stochastic coordinate descent. Our own work [3, 46] proves the convergence of lock-free execution for stochastic gradient descent and stochastic coordinate descent.

2.3.2 The DeepDive Approach and The Performance of PDD

The DeepDive Approach. The statistical inference and learning engine in DeepDive [4] is built upon the challenge of designing a high-performance statistical inference and learning engine on a single machine [4, 6, 7, 46]. Compared to traditional work, the main novelty of DeepDive is that it considers *both* hardware efficiency and statistical efficiency for executing an inference and learning task.

Hardware Efficiency. DeepDive takes into consideration the architecture of modern non-uniform memory access (NUMA) machines. A NUMA machine usually contains multiple nodes (sockets), where each sockets contains multiple CPU cores. To achieve high hardware efficiency, it is useful to decrease the communication across different NUMA nodes.

Statistical Efficiency Pushing hardware efficiency to the extreme might cause statistical efficiency to suffer because the lack of communication between nodes could decrease the rate of convergence of a statistical inference and learning algorithm. DeepDive takes advantage of theoretical results of model averaging [45] and lock-free execution [7, 46].

Performance of Statistical Inference and Learning. DeepDive enables PDD's ability to run statistical inference and learning efficiently. For example, on the whole corpus, the factor graph contains more than 0.2 billion random variables and 0.3 billion factors. On this factor graph, DeepDive is able to run Gibbs sampling on a machine with 4 sockets (10 core per sockets), and we find that we can generate 1,000 samples for all 0.2 billion random variables in 28 minutes.

References

[1] Heim, N., Knope, M. & Payne, J. L. Cope's rule in solitary marine bilaterian animals across the past 540 million years (In Preparation).

[2] Kumar, A., Niu, F. & Ré, C. Hazy: making it easier to build and maintain big-data analytics. *Commun. ACM* **56**, 40–49 (2013).

[3] Niu, F., Ré, C., Doan, A. & Shavlik, J. Tuffy: Scaling up statistical inference in markov logic networks using an rdbms. *Proc. VLDB Endow.* **4**, 373–384 (2011). URL `http://dl.acm.org/citation.cfm?id=1978665.1978669`.

[4] Zhang, C. & Ré, C. Dimmwitted: A study of main-memory statistical analytics. *ArXiv e-print.* (2013).

[5] Niu, F., Zhang, C., R, C. & Shavlik, J. W. Elementary: Large-scale knowledge-base construction via machine learning and statistical inference. *Int. J. Semantic Web Inf. Syst.* **8**, 42–73 (2012). URL `http://dblp.uni-trier.de/db/journals/ijswis/ijswis8.html#NiuZRS12`.

[6] Zhang, C. & Ré, C. Towards high-throughput gibbs sampling at scale: A study across storage managers. In *Proceedings of the 2013 ACM SIGMOD International Conference on Management of Data*, SIGMOD '13, 397–408 (ACM, New York, NY, USA, 2013). URL `http://doi.acm.org/10.1145/2463676.2463702`.

[7] Niu, F., Recht, B., Re, C. & Wright, S. J. Hogwild: A lock-free approach to parallelizing stochastic gradient descent. In *NIPS*, 693–701 (2011).

[8] Krishnamurthy, R. *et al.* Systemt: A system for declarative information extraction. *SIGMOD Rec.* **37**, 7–13 (2009). URL `http://doi.acm.org/10.1145/1519103.1519105`.

[9] Shen, W., Doan, A., Naughton, J. F. & Ramakrishnan, R. Declarative information extraction using datalog with embedded extraction predicates. In *Proceedings of the 33rd International Conference on Very Large Data Bases*, VLDB '07, 1033–1044 (VLDB Endowment, 2007). URL `http://dl.acm.org/citation.cfm?id=1325851.1325968`.

[10] Suchanek, F. M., Sozio, M. & Weikum, G. Sofie: A self-organizing framework for information extraction. In *Proceedings of the 18th International Conference on World Wide Web*, WWW '09, 631–640 (ACM, New York, NY, USA, 2009). URL `http://doi.acm.org/10.1145/1526709.1526794`.

[11] Nakashole, N., Theobald, M. & Weikum, G. Scalable knowledge harvesting with high precision and high recall. In *Proceedings of the Fourth ACM International Conference on Web Search and Data Mining*, WSDM '11, 227–236 (ACM, New York, NY, USA, 2011). URL `http://doi.acm.org/10.1145/1935826.1935869`.

[12] Etzioni, O. *et al.* Web-scale information extraction in knowitall: (preliminary results). In *Proceedings of the 13th International Conference on World Wide Web*, WWW '04, 100–110 (ACM, New York, NY, USA, 2004). URL `http://doi.acm.org/10.1145/988672.988687`.

[13] Yates, A. *et al.* Textrunner: Open information extraction on the web. In *Proceedings of Human Language Technologies: The Annual Conference of the North American Chapter of the Association for Computational Linguistics: Demonstrations*, NAACL-Demonstrations '07, 25–26 (Association for Computational Linguistics, Stroudsburg, PA, USA, 2007). URL `http://dl.acm.org/citation.cfm?id=1614164.1614177`.

[14] Banko, M., Cafarella, M. J., Soderland, S., Broadhead, M. & Etzioni, O. Open information extraction from the web. In *Proceedings of the 20th International Joint Conference on Artifical Intelligence*, IJCAI'07, 2670–2676 (Morgan Kaufmann Publishers Inc., San Francisco, CA, USA, 2007). URL `http://dl.acm.org/citation.cfm?id=1625275.1625705`.

[15] Kasneci, G., Ramanath, M., Suchanek, F. & Weikum, G. The yago-naga approach to knowledge discovery. *SIGMOD Rec.* **37**, 41–47 (2009). URL `http://doi.acm.org/10.1145/1519103.1519110`.

[16] Betteridge, J. *et al.* Toward never ending language learning. In *AAAI Spring Symposium: Learning by Reading and Learning to Read*, 1–2 (2009).

[17] Carlson, A. *et al.* Toward an architecture for never-ending language learning. In *AAAI* (2010).

[18] Zhu, J., Nie, Z., Liu, X., Zhang, B. & Wen, J.-R. Statsnowball: A statistical approach to extracting entity relationships. In *Proceedings of the 18th International Conference on World Wide Web*, WWW '09, 101–110 (ACM, New York, NY, USA, 2009). URL `http://doi.acm.org/10.1145/1526709.1526724`.

[19] Poon, H. & Domingos, P. Joint inference in information extraction. In *Proceedings of the 22Nd National Conference on Artificial Intelligence - Volume 1*, AAAI'07, 913–918 (AAAI Press, 2007). URL `http://dl.acm.org/citation.cfm?id=1619645.1619792`.

[20] Hearst, M. A. Automatic acquisition of hyponyms from large text corpora. In *Proceedings of the 14th Conference on Computational Linguistics - Volume 2*, COLING '92, 539–545 (Association for Computational Linguistics, Stroudsburg, PA, USA, 1992). URL `http://dx.doi.org/10.3115/992133.992154`.

[21] Li, Y., Reiss, F. R. & Chiticariu, L. Systemt: A declarative information extraction system. In *Proceedings of the 49th Annual Meeting of the Association for Computational Linguistics: Human Language Technologies: Systems Demonstrations*, HLT '11, 109–114 (Association for Computational Linguistics, Stroudsburg, PA, USA, 2011). URL `http://dl.acm.org/citation.cfm?id=2002440.2002459`.

[22] Domingos, P. & Lowd, D. *Markov Logic: An Interface Layer for Artificial Intelligence*. Synthesis Lectures on Artificial Intelligence and Machine Learning (Morgan & Claypool Publishers, 2009).

[23] Jaynes, E. *Probability Theory: The Logic of Science* (Cambridge Univ. Press, 2003).

[24] Buskirk, E. V. How the netix prize was won. *Wired* (2009).

[25] Ferrucci, D. A. *et al.* Building watson: An overview of the deepqa project. *AI Magazine* **31**, 59–79 (2010). URL `http://dblp.uni-trier.de/db/journals/aim/aim31.html#FerrucciBCFGKLMNPSW10`.

[26] Mintz, M., Bills, S., Snow, R. & Jurafsky, D. Distant supervision for relation extraction without labeled data. In *Proceedings of the Joint Conference of the 47th Annual Meeting of the ACL and the 4th International Joint Conference on Natural Language Processing of the AFNLP: Volume 2 - Volume 2*, ACL '09, 1003–1011 (Association for Computational Linguistics, Stroudsburg, PA, USA, 2009). URL `http://dl.acm.org/citation.cfm?id=1690219.1690287`.

[27] Finkel, J. R., Kleeman, A. & Manning, C. D. Efficient, feature-based, conditional random field parsing. In *ACL*, 959–967 (2008).

[28] Govindaraju, V., Zhang, C. & Ré, C. Understanding tables in context using standard nlp toolkits. In *ACL (2)*, 658–664 (2013).

[29] Min, B., Grishman, R., Wan, L., Wang, C. & Gondek, D. Distant supervision for relation extraction with an incomplete knowledge base. In *NAACL* (2013).

[30] McCallum, A. Joint inference for natural language processing. In *Proceedings of the Thirteenth Conference on Computational Natural Language Learning*, CoNLL '09, 1–1 (Association for Computational Linguistics, Stroudsburg, PA, USA, 2009). URL `http://dl.acm.org/citation.cfm?id=1596374.1596376`.

[31] Poon, H. & Vanderwende, L. Joint inference for knowledge extraction from biomedical literature. In *Human Language Technologies: The 2010 Annual Conference of the North American Chapter of the Association for Computational Linguistics*, HLT '10, 813–821 (Association for Computational Linguistics, Stroudsburg, PA, USA, 2010). URL `http://dl.acm.org/citation.cfm?id=1857999.1858122`.

[32] Collobert, R. & Weston, J. A unified architecture for natural language processing: Deep neural networks with multitask learning. In *Proceedings of the 25th International Conference on Machine Learning*, ICML '08, 160–167 (ACM, New York, NY, USA, 2008). URL `http://doi.acm.org/10.1145/1390156.1390177`.

[33] Collobert, R. *et al.* Natural language processing (almost) from scratch. *Journal of Machine Learning Research* **12**, 2493–2537 (2011). URL `http://leon.bottou.org/papers/collobert-2011`.

[34] Mitchell, T. M. *Machine Learning* (McGraw-Hill, USA, 1997).

[35] Oracle R Enterprise. `docs.oracle.com/cd/E27988_01/doc/doc.112/e26499.pdf`.

[36] Hellerstein, J. M. & et al. The MADlib analytics library: Or MAD skills, the SQL. *PVLDB* 1700–1711 (2012).

[37] Ghoting, A. & et al. SystemML: Declarative machine learning on MapReduce. In *ICDE* (2011).

[38] Apache Mahout. `mahout.apache.org`.

[39] Sparks, E. & et al. MLI: An API for distributed machine learning. In *ICDM*, 1187–1192 (2013).

[40] Low, Y. & et al. Distributed GraphLab: A framework for machine learning in the cloud. *PVLDB* 716–727 (2012).

[41] Kyrola, A., Blelloch, G. & Guestrin, C. Graphchi: Large-scale graph computation on just a pc. In *Proceedings of the 10th USENIX Conference on Operating Systems Design and Implementation*, OSDI'12, 31–46 (USENIX Association, Berkeley, CA, USA, 2012). URL `http://dl.acm.org/citation.cfm?id=2387880.2387884`.

[42] Chafi, H. *et al.* A domain-specific approach to heterogeneous parallelism. In *PPOPP*, 35–46 (2011).

[43] Sujeeth, A. K. & et al. OptiML: An Implicitly Parallel Domain-Specific Language for Machine Learning. In *ICML*, 609–616 (2011).

[44] Tsitsiklis, J., Bertsekas, D. & Athans, M. Distributed asynchronous deterministic and stochastic gradient optimization algorithms. *IEEE Transactions on Automatic Control* 803–812 (1986).

[45] Zinkevich, M. & et al. Parallelized stochastic gradient descent. In *NIPS*, 2595–2603 (2010).

[46] Liu, J. & et al. An asynchronous parallel stochastic coordinate descent algorithm. *ICML* (2014).

[47] Bradley, J. K., Kyrola, A., Bickson, D. & Guestrin, C. Parallel coordinate descent for l1-regularized loss minimization. In *ICML*, 321–328 (2011).
